# Genotype by environment interaction analysis for resistance against powdery mildew and yellow rust in some promising exotic wheats

**DOI:** 10.1186/s12870-025-06788-0

**Published:** 2025-07-01

**Authors:** Amritpal Mehta, Daisy Basandrai, Harneet Kaur, Jaspal Kaur, Ashwani Kumar Basandrai, Umer Basu, Sukhwinder Singh, Waleed A. A. Alsakkaf, Hayssam M. Ali

**Affiliations:** 1 Department of Plant Pathology, CSK Himachal Pradesh Agricultural University, Palampur, 176062 India; 2 ICAR-National Institute of Biotic Stress Management, Baronda, Raipur, Chhattisgarh 493225 India; 3 Department of Genetics and Plant Breeding, CSK Himachal Pradesh Agricultural University, Palampur, 176062 India; 4 Department of Plant Breeding and Genetics, Punjab Agricultural University, Ludhiana, Punjab 141004 India; 5 College of Plant Protection, Northwest A & F University, Yangling, Shaanxi 712100 China; 6 Key Laboratory of Integrated Pest Management On Crops in North-Western Loess Plateau, Ministry of Agriculture and Rural Affairs, Yangling, Shaanxi 712100 China; 7 Key Laboratory of Plant Protection Resources & Pest Management, Ministry of Education, Yangling, Shaanxi 712100 China; 8 USDA-ARS, Subtropical Horticulture Research Station, 13601 Old Cutler Road, Miami, Fl 33158 USA; 9 Department of Botany and Microbiology, College of Science, King Saud University, Saudi Arabia 11451 Riyadh,

**Keywords:** Powdery mildew, Yellow rust, GGE biplot, AMMI, AMMI stability value, Principal Components, Slow mildewing resistance, Exotic germplasm

## Abstract

**Background:**

Powdery mildew (PM), caused by *Blumeria graminis* f. sp. *tritici*, and yellow rust (YR), caused by *Puccinia striiformis* f. sp. *tritici*, constitute significant threats to wheat production, resulting in both qualitative and quantitative losses. Although fungicides can effectively manage these diseases, their application introduces environmental and health risks and foster the development of pathogen resistance. The development and implementation of wheat genotypes that exhibit resistance to PM and YR present a sustainable, cost-effective, and environmentally responsible alternative to chemical treatments.

**Results:**

In the present study, influence of environmental factors and genotype by-environment interaction (GEI) was evaluated on 142 wheat genotypes for PM and YR across four and three geographically diverse hotspot locations, respectively. The AMMI analysis of variance revealed that GEI and genotype (G) accounted for most of the variation observed for PM and YR. Twenty genotypes were moderately resistant to PM at seedling stage. Notably, ten genotypes demonstrated high resistance to PM, while 37 were identified as resistant to YR. Furthermore, 30 genotypes exhibited slow mildewing resistance to PM at the adult plant stage. The combined analysis utilizing AMMI and GGE biplots indicated that the genotypes Pollmer/CTY88.547, Syros, and Talent (*Pm5* + *?*) portrayed the highest level of combined resistance to both PM and YR across the evaluated locations. Additionally, the environments Kukumseri 2016 (E2 and En1) were the most effective for testing and selecting superior wheat genotypes for resistance to PM and YR, respectively.

**Conclusion:**

Integrating the strength of AMMI and GGE approaches enhances the accuracy of wheat genotype selection in multi- environment trials. Methods used showed strong agreement in identifying wheat genotypes resistant to PM and YR when facing diverse environmental factors.

**Supplementary Information:**

The online version contains supplementary material available at 10.1186/s12870-025-06788-0.

## Background

Wheat (*Triticum* spp.) is a widely grown cereal crop and a staple food for over one-third of the world's population. Its production is severely affected by various biotic and abiotic factors, including climate change and crop related management [1–5]. Among various biotic factors, powdery mildew (PM) and yellow rust (YR) caused by *Blumeria graminis* f. sp. *tritici* (*Bgt*) (syn*. Erysiphe graminis* (DC) f. sp. *tritici* EJ Marchal) and *Puccinia striiformis* Westend. f. sp. *tritici* Erikss. (*Pst*), respectively are widespread and devastating wheat diseases, significantly impacting global wheat production. These diseases are widely prevalent in India, especially in the Northern Hill Zone and the North Western Plain Zone [6, 7]. The yield losses ranging from 13–100% and 10–70%, have been reported under low to severe epidemics of PM and YR, respectively [8, 9]. Therefore, combating the disease pressure of both the diseases and to reduce the associated yield losses, warrants developing cost effective, eco- and farmer friendly, and long-term sustainable management strategies. Foliar fungicide sprays are certainly the effective management options; however, the long-term use of a particular fungicide can lead to the development of resistance in pathogen populations and the accumulation of toxic residues, endangering human health, and soil, water and the environment [10, 11]. Under these circumstances, use of resistant varieties offers the most sustainable alternative for reducing the losses caused by both the diseases. Nevertheless, a considerable proportion of the widely cultivated commercial wheat varieties in epidemiologically significant regions of India are susceptible to these diseases [2, 7]. Furthermore, the resistance exhibited by existing varieties is frequently partial and short-lived. In the light of these challenges, it is essential to identify diverse donor varieties. Incorporating such varieties into breeding programs represents a critical and urgent responsibility for plant breeders and pathologists aiming to develop robust wheat varieties resistant to PM and YR. Stable resistance in host plants is evaluated based on how consistently a plant maintains its original resistance level over time against a broad spectrum of pathogen races or pathotypes [12]. To assess this stability, host genotypes must be repeatedly tested across different environments, either through multi-location trials within a single year or through trials conducted at the same location over several years [13]. Genotype by environment interaction (GEI) plays a crucial role in crop improvement, as it complicates the comparison of genotypes across test environments and makes it more difficult to define breeding objectives. Overcoming these challenges requires a deeper understanding of plant adaptation, particularly the variations in performance and the nature of GEI [14, 15]. Because GEI makes it harder to select superior genotype, plant breeders often rely on multi-environment trials (MET) to evaluate and interpret the complexities associated with GEI [15, 16]. Understanding the role of environmental factors (mainly locations; E), genotypes (G) and their interaction (GEI) in relation to the pathosystem and host genotype stability across different locations is imperative for an effective resistance breeding program [1, 17]. A variety of tools have been employed to characterize environments and distinguish stable and unstable genotypes across different crops. Regression based methods and multivariate analyses are the most widely used in cultivar release programs to assess stability. Multivariate approaches are particularly valuable, as they enable the integration of genotype responses across diverse environments, providing a more comprehensive understanding of stability and adaptability [16]. Among them, the additive main effects and multiplicative interaction (AMMI) model and genotype main effect and genotype-by-environment interaction (GGE) biplot analyses are better methods for the identification of stable and adaptable genotypes for specific or diverse environments and identification of hot spot location for resistance evaluation [1, 16, 18, 19]. The AMMI model collectively considers G, E, and their interaction with each other (G × E) as individual parameters [20], overcomes structural variations among the genotypes across environments thereby improving data precision. GGE biplot method simultaneously and graphically analyses the main effect of G and its interaction with the E [17, 21, 22]. In the present study, GGE biplot and AMMI analysis were used to identify the genotypes with stable resistance and to determine ideal test environments for screening some promising indigenous and exotic wheat genotypes against PM and YR disease.

## Materials and methods

The study included 142 wheat genotypes obtained from ICAR-IIWBR in Karnal, CIMMYT in Mexico, and Punjab Agricultural University in Ludhiana. Detailed information regarding their pedigree, parentage, origin, and the genes for powdery mildew (*Pm*) and yellow rust (*Yr*) is provided in Table 2. The genotypes were assessed at the adult plant stage (APS) at the Rice and Wheat Research Centre (RWRC), (Malan 2016–17) (E1), Highland Agricultural Research and Extension Centre (HAREC), (Kukumseri 2016) (E2) during summer, CSKHPKV, Palampur (Palampur 2016–17) (E3) and (Palampur 2017–18) (E4) growing seasons for PM. Evaluations for YR, were conducted at the HAREC, (Kukumseri 2016) (En1) during summer, Research Station of Punjab Agricultural University, (Keylong 2017) (En2) during summer and RWRC, (Malan 2016–17) (En3) (Table 2).

### Evaluation against powdery mildew (PM) at seedling stage

Seedlings of each line along with susceptible check Lehmi were grown in galvanized iron trays under polyhouse at RWRC, Malan following recommended agronomic practices [23] and were irrigated as per need. Seedlings of each entry at one or two leaf stage (10 days old) were inoculated using fresh and viable conidial inoculum of local field isolate of *Bgt*, mass-multiplied on susceptible cultivars such as Agra Local, HPW 155, Lehmi etc. The avirulence/virulence formula of the isolate was: *Pm1c*, *Pm2*, *Pm3b*, *Pm4a*, *Pm2 mldb*, *Pm1* + *2* + *9* + *12*/*Pm1a*, *Pm3a*, *Pm3c*, *Pm3 d*, *Pm3f*, *Pm5a*, *Pm6*, *Pm8*, *Pm10*, *Pm12*, *Pm17*, *Pm25*, *Pm10* + *15*. The inoculated seedlings were placed in a polyhouse under natural light, with incubation temperature maintained at a minimum of 5 ± 3 °C and a maximum of 20 ± 5 °C. The data on infection-type (IT) were recorded 10 days post-inoculations, following the modified 0–4 scale proposed by Smith and Blair [24], once the susceptible cultivar exhibited ‘IT = 4’ response.

### Evaluation against powdery mildew (PM) at adult plant stage

Established agricultural practices were followed to cultivate the test genotypes in single-row plots, each measuring 1 m in length [25]. The susceptible check variety, Lehmi, was sown after every tenth test genotype. To promote propagation and dissemination of inoculum, the experimental plot was bordered by infector rows of either PBW 343 or Lehmi. Disease symptoms manifest earlier and more severely in the susceptible check varieties. To dislodge the conidia, which serve as inoculum for infecting healthy plants, these plants were gently tapped with a wooden stick in the evening, allowing the conidia to be dispersed by the wind. Data about disease severity (expressed as a percentage) was periodically recorded from randomly selected five plants in each test genotype. The data were collected using the modified scale of Mayee and Datar, [26] and was subsequently utilized to calculate the Area Under the Disease Progress Curve (AUDPC), the per unit disease increase (r), and the relative Area Under the Disease Progress Curve (rAUDPC). These calculations were critical for identifying genotypes that exhibit slow mildewing resistance. The AUDPC was computed following the formula proposed by Shaner and Finney [27]:

$$AUDPC=\sum\limits_{i=1}^{n-1}(x_i+x_{i+1})/2\times(t_{i+1}-t_i)$$

where *yi* represents disease (%) at the *i*th observation, *ti* denotes the time (weekly interval) at the *i*th observation, and *n* represents the observations number.

The infection rate (r) was computed as per Vander Plank [28].

$$\text{r}=\frac{2.3}{{t}^{2 }- {t}^{1}} x \text{log}10 \frac{{x}^{2} (1-{x}^{1})}{{x}^{1} (1-{x}^{2})}$$

where, *x*^1^ = The proportion of infected tissues at a given time t.^1^

*x*^2^ = The proportion of infected tissues at a given time t.^2^

*t*^2^-*t*^1^ = time interval.

Relative AUDPC (rAUDPC) was calculated as per Ma and Singh [29] where,

$$\text{rAUDPC}=\frac{\text{AUDPC of test genotype}}{\text{AUDPC of susceptible check var}.} x 100$$

### Evaluation for yellow rust (YR)

All test locations, except Palampur are recognized as hot spots for YR. Artificial epiphytotic conditions of YR were created to prevent the disease from escaping. The inoculum, consisting of the mixture of prevailing *Pst* races in the region, was obtained from ICAR-IIWBR, Regional Station, Shimla and mass-multiplied on the variety PBW 343. A suspension containing 1 × 10^6^ uredospores/ml of water was sprayed onto the test genotypes, as well as the susceptible check varieties planted after every tenth test row and around the experimental field. Additionally, a mixture of local *Pst* field populations collected from farmers’ fields was applied at all locations, particularly at Kukumseri and Keylong*.* Disease data were recorded using the scale outlined by Roelfs et al. [30], while percentage severity was assessed according to the modified Cobb’ s scale [31].

### Statistical analysis

The additive main effects and multiplicative interaction (AMMI) analysis of variance was computed using the ‘metan’ package [32] in R-Studio version 4.0.5 [33].

The AMMI model combines ANOVA and principal component analysis (PCA), describing the summative effects of G, E and GEI. The GEI was partitioned among the first and second interaction principal components axes (IPC1 and IPC2). IPC1 (X-axis) indicates resistance level, while IPC2 (Y-axis) represents genotype stability [34]. ANOVA was used to analyse data with main effects of G and E without their interaction, and PCA was integrated using standardized residuals. The AMMI model followed Gauch and Zobel [35] and Yan et al. [36], to assess the relationships between G, E, and GEI for the PM and YR resistance.

$$\overline{y}ij= \mu + {g}_{i}+ {e}_{i}+ \sum_{k=1}^{n}{\lambda }_{k}{\alpha }_{ik}{\gamma }_{ij}+ {r}_{ij}+ {\rho }_{ij}$$

where *Y*_*ij*_ is the disease severity of the *i-*th genotype in the *j-*th environment; *μ* is the grand mean; *g*_*i*_ and *e*_*j*_ represent the genotype and environment deviations from the grand mean; *λ*_*k*_ is the square root of the eigenvalue of the *k-*th IPCA analysis axis; *α*_*ik*_ and *γ*_*ij*_ are the principal component scores for the *k-*th IPCA axis of the *i-*th genotype and the *j-*th environment; *n* considered as number of principal components retained in the model; *r*_*ij*_ is the effect of the *jth* block nested within the *i-*th replica; and *ρ*_*ij*_ is the deviation from the model.

AMMI Stability Value (ASV) was calculated using the formula developed by Purchase et al. [19]:

$$\text{ASV}=\sqrt{[ \frac{SS IPCA1}{SS IPCA2} \left(IPCA1 score\right){]}^{2}+(IPCA2 score{)}^{2}}$$

where, SS IPCA1 is sum of squares of interaction principal component analysis 1 (IPCA1) and SS IPCA2 is sum of squares of IPCA2.

In the AMMI analysis, model diagnosis and evaluation of predictive accuracy specifically the ability to accurately estimate true means were performed according to the methodologies outlined by Ebdon and Gauch [37] and Gacuh [38]. The actual root mean square predictive differences (RMSPD) was computed using the metan’ package [32] in R-Studio version 4.0.5 [33].

To determine the winning genotypes in various test environments and to identify mega-environments (MEs), a “which-won-where?” view of GGE biplot was generated by genotype focused singular-value partitioning (SVP = 1) [39]. The correlation between various environments was determined based on the cosine of the angles between the environment vectors, determined through environment-focused SVP (SVP = 2) [40]. Moreover, “discriminating power vs. representativeness” view of the GGE biplot was created to evaluate the test locations by symmetrical scale (SVP = 3). In this view, the “ideal” test environment is one that can discriminate between the genotypes and is also representative of the ME [41].

The GGE model was used to assess the stability of genotypes across locations, described by the equation:

$$Yij- \mu -\beta j= \lambda 1\xi il\eta lj+ \varepsilon ij$$

where *Yij* represents the mean of PM and YR severity for the *i-*th genotype grown in *j-*th environment, *μ* is the grand mean for all environment, *βj* is the main effect of the *j*-th environment, *η* is the singular value, *λ* and ξ are the singular vectors for genotype and environment for n = 1, 2, 3,...., respectively, and *εij* is the residual effect for each genotype-environment combination that is not explained by the principal components PC1 and PC2 [Yan et al., 2000]. Furthermore, the genotypic means relative to the principal components were graphically displayed in GGE (genotype + genotype by environment) biplots using the ‘metan’ package [32] in R-Studio version 4.0.5 [33].

Differences in mean PM and YR scores across the test locations were compared using the T-test. Furthermore, bar graphs were generated through the ‘rstatix’ and ‘ggpubr’ packages in R-Studio version 4.0.5 [33].

## Results

### AMMI analysis of variance

**Fig. 1:**
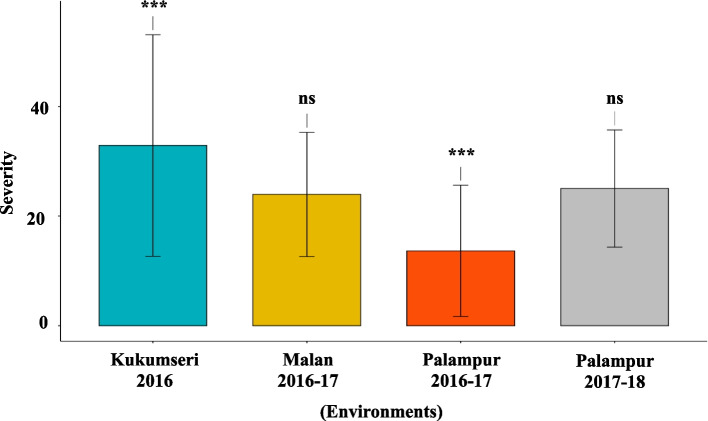
Mean PM disease severity for the four test locations. The significance of differences for PM disease severity among test locations was calculated using T-test. *** = *p* < 0.001; ns = non-significant

**Fig. 2:**
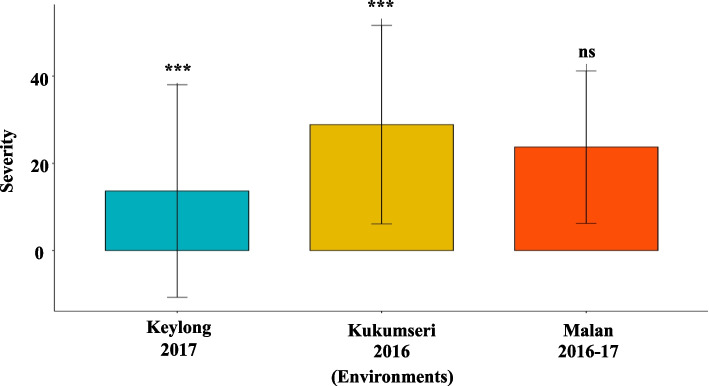
Mean YR disease severity for the three test locations. The significance of differences for YR disease severity among test locations was calculated using T-test. *** = *p* < 0.001; ns = non-significant

**Fig. 3:**
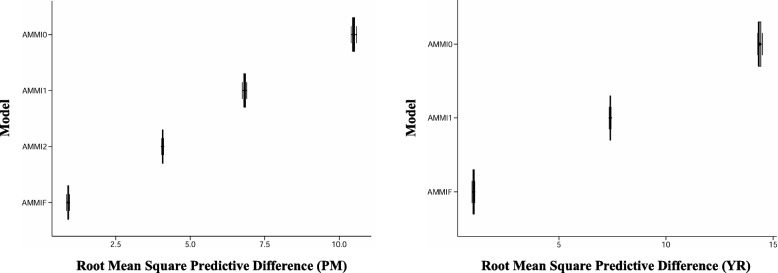
Figure showing RMSPD estimates of different prediction models tested for powdery mildew (PM) and yellow rust (YR)

The results revealed significant differences (*P* < *0.001*) for disease severity among 142 wheat genotypes across 4 and 3 environments against PM and YR, respectively (Figs. 1 and 2). This indicated that PM and YR severity were highly influenced by G, E and GEI. GEI, G, and E contributed 44.64 & 40.27%, 36.25 & 51.94%, and 19.11 and 7.78% of the total variation for PM and YR, respectively (Table 1). Moreover, AMMI analysis revealed that GEI was significantly explained by the first three principal components (PCs) for PM and the first two PCs for YR. For PM, the PC1 contributed 58.17% toward the total GEI with 143 degree of freedom (df) while, PC2 and PC3 contributed 27.36%, and 14.46% with 141 and 139 df, respectively. PC1 contributed 73.77% toward the total GEI for YR while, PC2 contributed 26.22% with 142 and 140 df, respectively (Table 1). To assess the accuracy of the AMMI models, the RMSPD was calculated for each. A lower RMSPD value indicated a more accurate model. Based on the RMSPD values presented in (Fig. 3), the AMMI F model is the most accurate for both the PM and YR datasets.

**Table 1:** Additive main effects and multiplicative interaction (AMMI) analysis of variance for PM and YR in wheat at 4 and 3, sites, respectively in North Western Himalayas (Himachal Pradesh)

Sources of Variations	DF	Sum of Square	Mean Square	Total Variation %	GEI Explained	GEI Cumulative	*P value*
**Powdery mildew (PM)**							
Environment	3	79902.07	26634.02	19.11			< *0.001*
Genotype	141	151622.20	1075.34	36.25			< *0.001*
Genotype x Environment	423	186693.10	441.35	44.64			< *0.001*
PC1	143	108607.80	759.50		58.17	58.17	< *0.001*
PC2	141	51082.97	362.28		27.36	85.54	< *0.001*
PC3	139	27003.36	194.27		14.46	100.00	< *0.001*
PC4	137	0.00	0.00		0.00	100.00	1
Residuals	1136	628.47	0.55				
		**Normality Test**					
Shapiro–Wilk		0.9542 (*P value* < 0.001)					
Anderson–Darling		16.82 (*P value* < 0.001)					
**Yellow rust (YR)**							
Environment	2	50755.17	25377.58	7.78			< *0.001*
Genotype	141	338808.40	2402.90	51.94			< *0.001*
Genotype x Environment	282	262705.00	931.58	40.27			< *0.001*
PC1	142	193806.70	1364.84		73.77	73.77	< *0.001*
PC2	140	68898.21	492.13		26.22	100.00	< *0.001*
PC3	138	0.00	0.00		0.00	100.00	1
Residuals	852	578.03	0.68				
		**Normality Test**					
Shapiro–Wilk		0.869 (*P value* < 0.001)					
Anderson–Darling		52.277 (*P value* < 0.001)					
**Correlation of PM and YR severity among genotypes**		−0.028^NS^					

*DF degree of freedom, *GEI* genotype by environment interaction, *NS *non-significant

### Discriminating ability and representativeness of environments

**Fig. 4:**
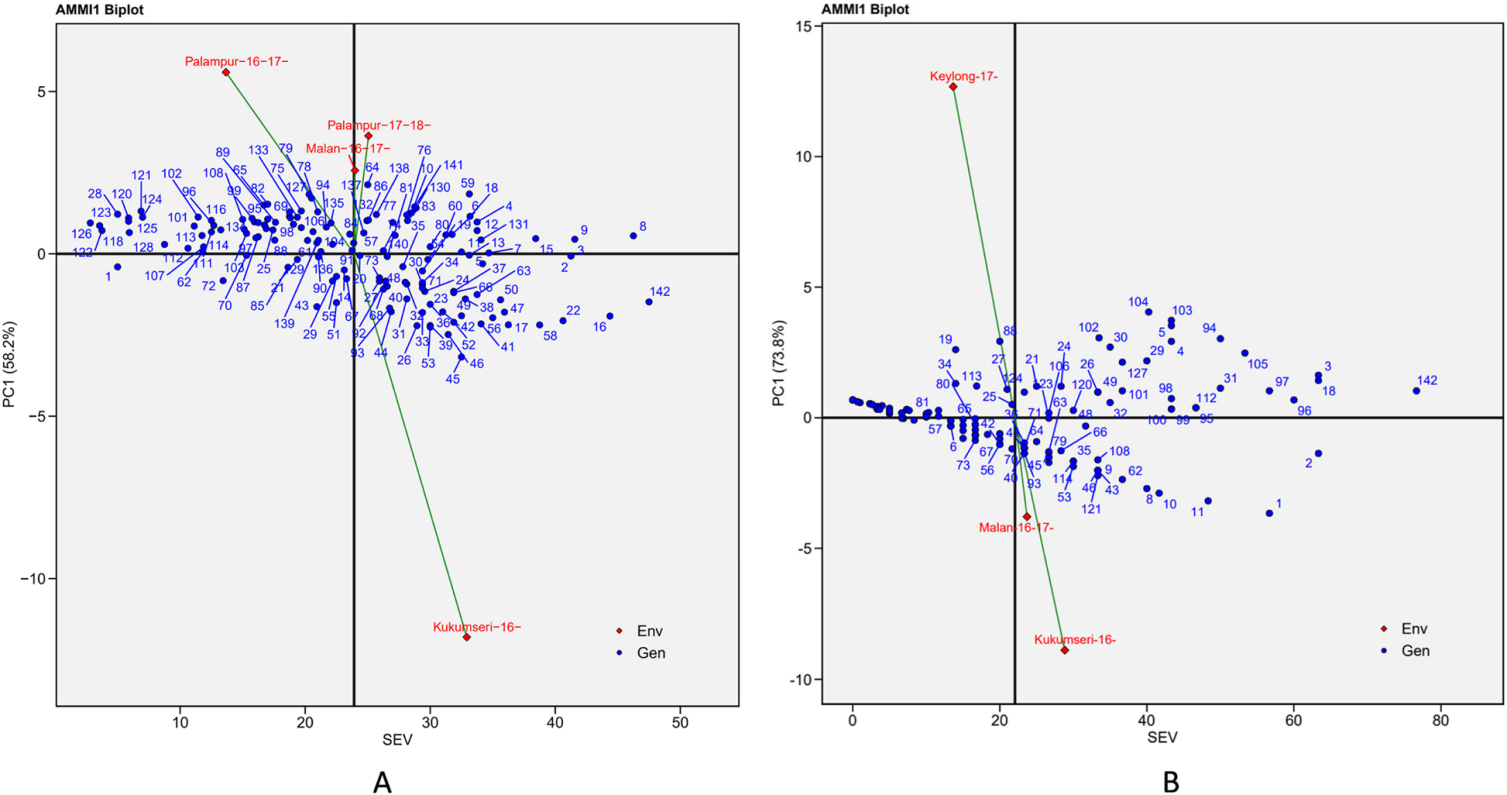
AMMI1 Biplot display for mean disease severity and IPCA 1 scores of the 142 wheat genotypes (G) tested across four and three environments for PM and YR, respectively. **A** Powdery mildew (PM) {Malan-16–17- = E1 (Malan 2016–17); Kukumseri-16- = E2 (Kukumseri 2016); Palampur-16–17- = E3 (Palampur 2016–17); Palampur-17–18- = E4 (Palampur 2017–18) and **B**) Yellow rust (YR) {Kukumseri-16- = En1 (Kukumseri 2016); Keylong-17- = En2 (Keylong 2017); Malan-16–17- = En3 (Malan 2016–17)} SEV = severity, Env = environment, Gen = genotype

AMMI 1 biplot showed the relationship of disease severity with genotypes under different environments, with disease severity on the abscissa and the respective IPC1 scores on the ordinate (Fig. 4A and B). Environment with IPCA-1 scores of zero or close to zero (either positive or negative) exhibited small interactions whereas, those with large IPCA-1 scores exhibited high interaction effect [15, 42]. In the current investigation, E3 and En3 with low IPCA-1 scores had little interactions for PM and YR, respectively, whereas a greater effect was observed in E4 for PM and En2 for YR (Table-S1). The disease expression was strongly influenced by the environment. Environments, E2 and E4 for PM and En1 and En3 for YR, positioned on the right side of the central axis, indicated that these environments were more favourable for disease expression and screening of genotypes against PM and YR, respectively. Contrarily, environments E1 and E3 for PM and En2 for YR were less favourable.

**Fig. 5:**
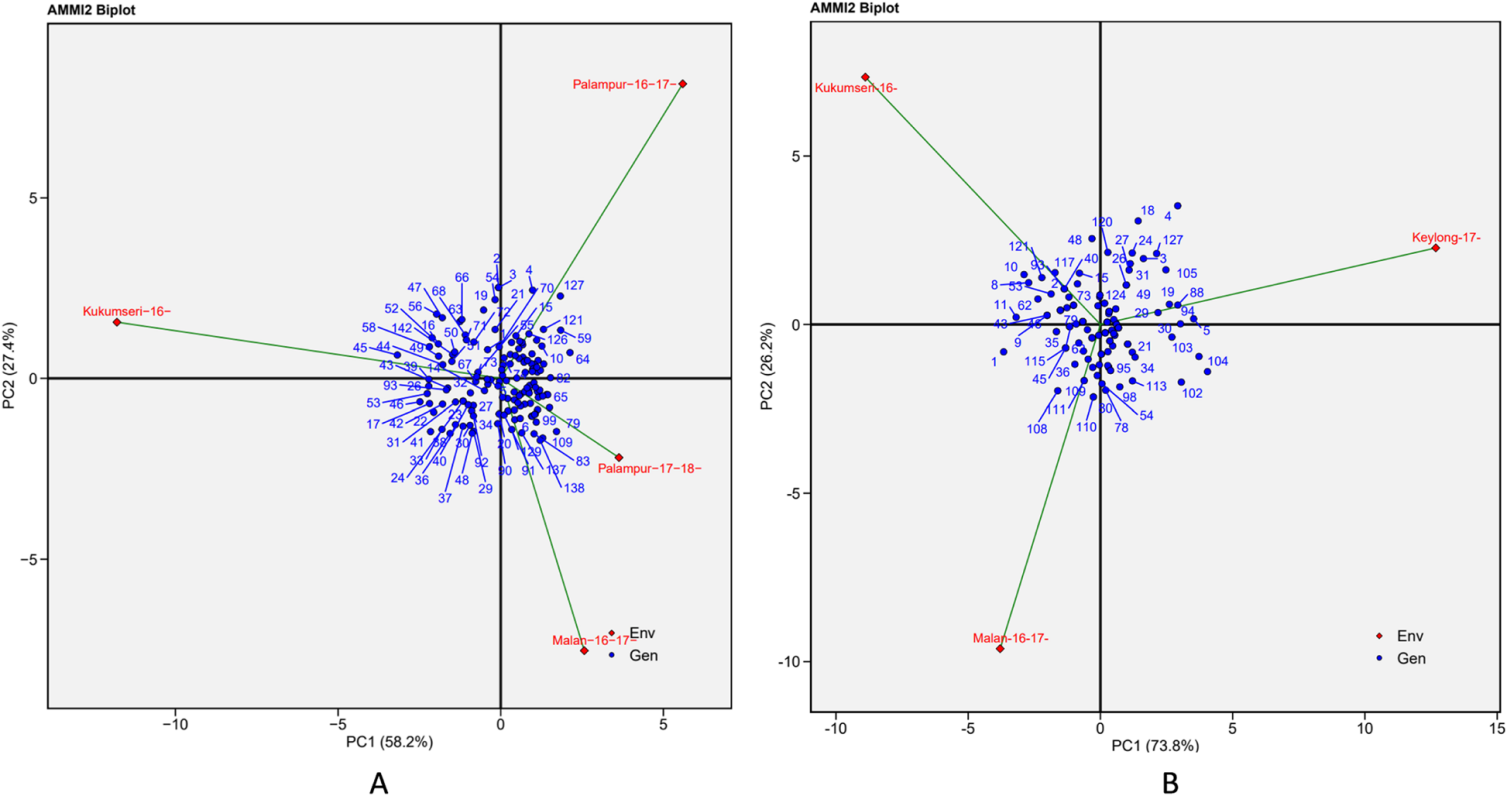
AMMI2 biplot for 142 genotypes and four environments for PM and three environments for YR display on the first and second principal component axis (IPAC 1 vs IPAC 2). **A**) Powdery mildew (PM) {Malan-16–17- = E1 (Malan 2016–17); Kukumseri-16- = E2 (Kukumseri 2016); Palampur-16–17- = E3 (Palampur 2016–17); Palampur-17–18- = E4 (Palampur 2017–18) and **B**) Yellow rust (YR) {Kukumseri-16- = En1 (Kukumseri 2016); Keylong-17- = En2 (Keylong 2017); Malan-16–17- = En3 (Malan 2016–17)} Env = environment, Gen = genotype

In AMMI2, environments which were placed near the origin, having low scores for IPCA1 and IPCA2 had small contribution to the GE interaction, but large contribution to the stability of genotypes. The AMMI 2 biplot revealed that environments E1, E2 and E3 for PM and En1, En2 and En3 for YR, were the most discriminating environments, indicating their significant influence on the variation in disease severity across the genotypes (Fig. 5A and B).

**Fig. 6:**
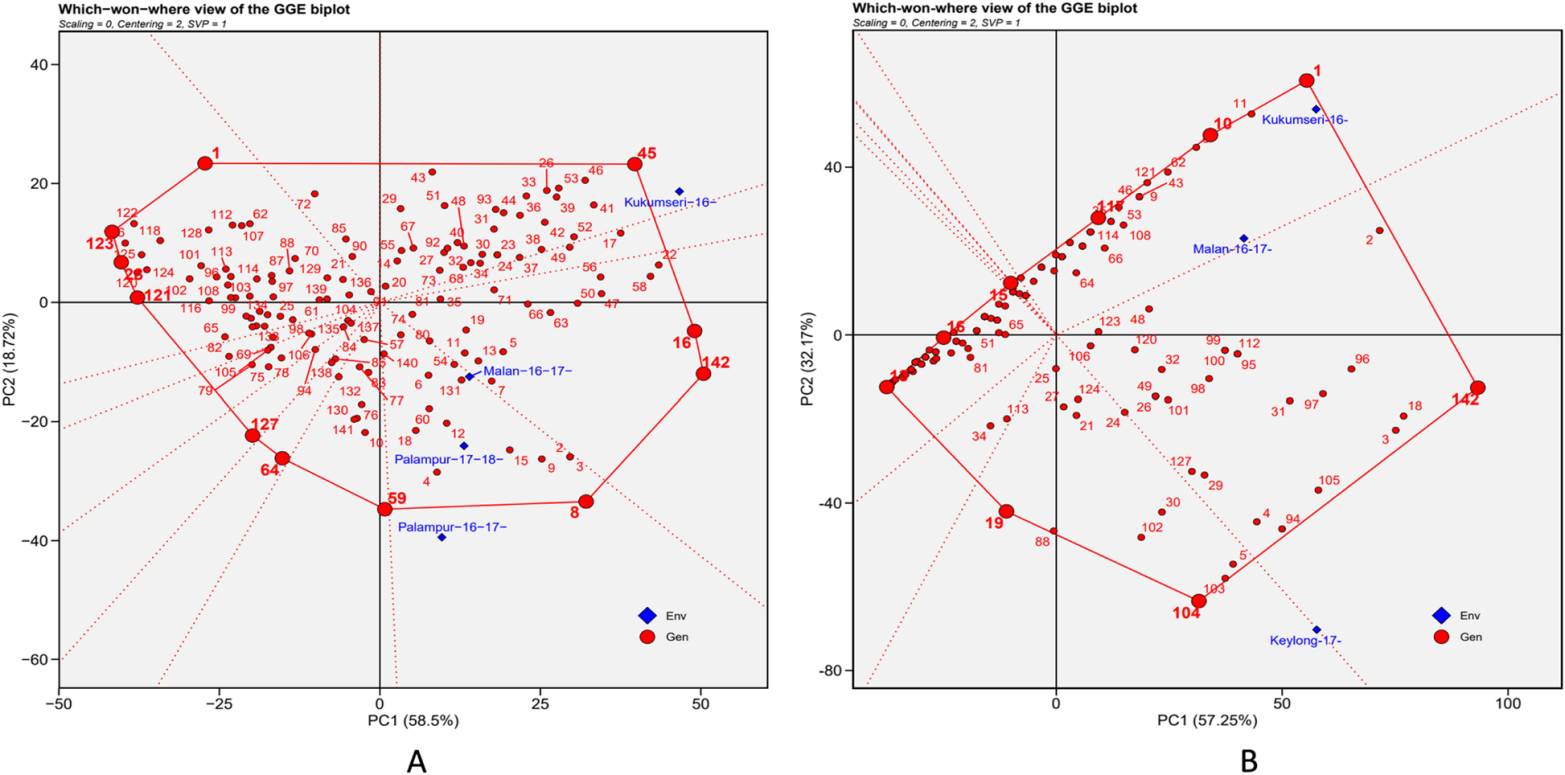
”Which-won-where” view of the GGE biplot of 142 wheat genotypes across 4 and 3 testing locations for PM and YR, respectively. There was no transformation of data (transform = 0), and data were centred by means of the environments (centring = 2). The biplot was based on genotype focused singular-value partitioning (SVP = 1). **A** Powdery mildew (PM) {Malan-16–17- = E1 (Malan 2016–17); Kukumseri-16- = E2 (Kukumseri 2016); Palampur-16–17- = E3 (Palampur 2016–17); Palampur-17–18- = E4 (Palampur 2017–18) and **B**) Yellow rust (YR) {Kukumseri-16- = En1 (Kukumseri 2016); Keylong-17- = En2 (Keylong 2017); Malan-16–17- = En3 (Malan 2016–17)} Env = environment, Gen = genotype

The GGE biplots analysis showed that PC1 (PM and YR disease) & PC2 (resistance stability) accounted for 58.50 & 57.25% and 18.72 & 32.17% of the total variation for PM and YR, respectively (Fig. 6A and B).

In biplot, the perpendicular red dashed lines divide the polygon into various sectors, aiding in the visualization of the Mega environments (MEs) [43]. The environmental layout in the biplot depicted the presence of 10 and 8 sector and two different MEs for PM and YR, respectively. The ME-I comprised one location E2 for PM and two locations En1 and En3 for YR whereas, ME-II encompassed three locations E1, E3 and E4 for PM and one location En2 for YR (Fig. 6A and B).

**Fig. 7:**
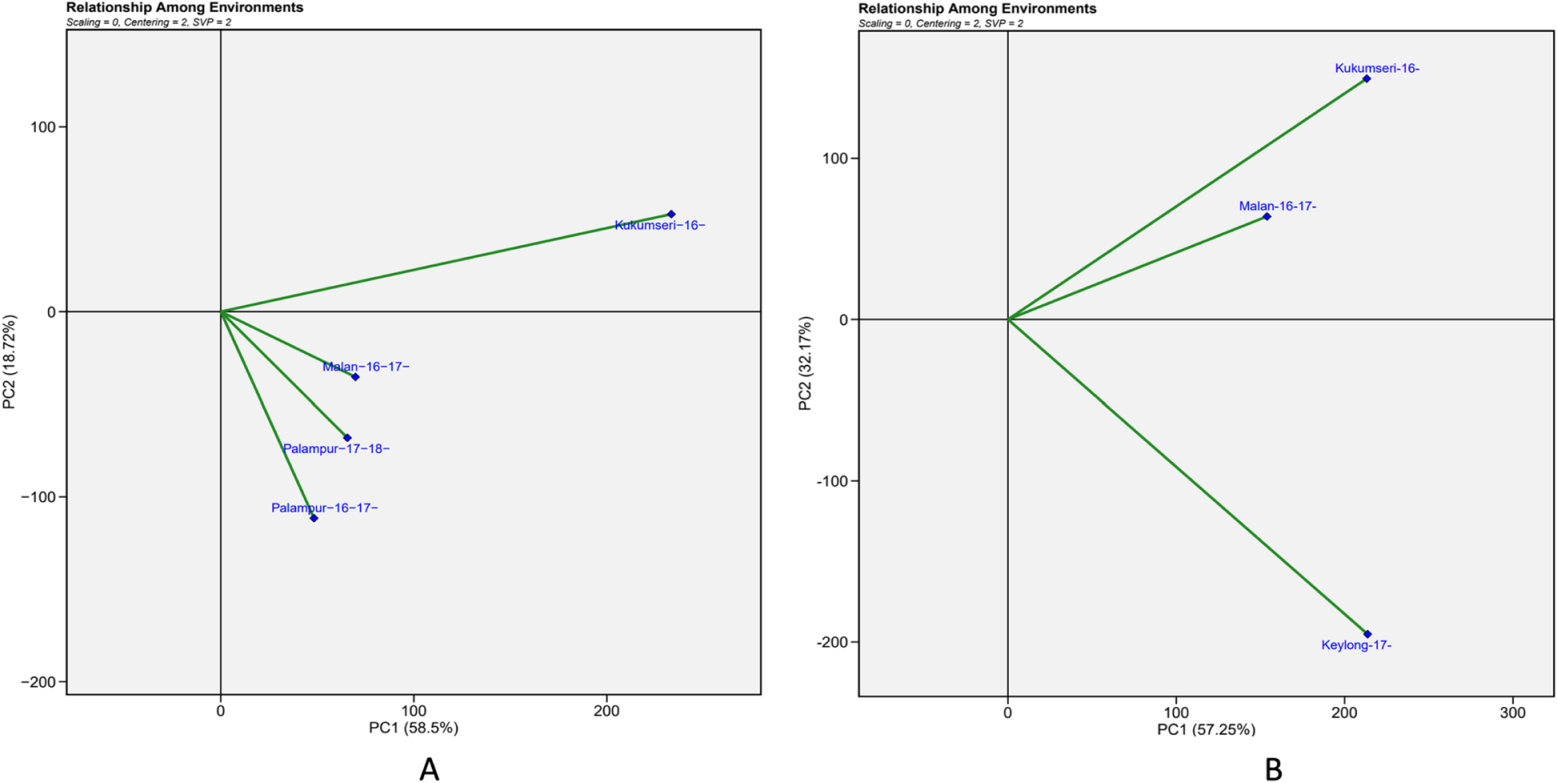
“Relationship among environments” view of the GGE biplot of 142 wheat genotypes across 4 and 3 testing locations for PM and YR, respectively. There was no transformation of data (transform = 0), and data were centred by means of the environments (centring = 2). The biplot was based on environment focused singular-value partitioning (SVP = 2). **A** Powdery mildew (PM) {Malan-16–17- = E1 (Malan 2016–17); Kukumseri-16- = E2 (Kukumseri 2016); Palampur-16–17- = E3 (Palampur 2016–17); Palampur-17–18- = E4 (Palampur 2017–18) and **B**) Yellow rust (YR) {Kukumseri-16- = En1 (Kukumseri 2016); Keylong-17- = En2 (Keylong 2017); Malan-16–17- = En3 (Malan 2016–17)}

In the “relationship among test environments” biplot, the smallest angle was observed between vectors of E3 and E4, as well as between E2 and E4 for PM and between En1 and En3 for YR, indicating the existence of positive correlation between them. This showed that genotypes performing the best at E3, and En1 can repeat the same performance at E4 and En2 for PM and YR, respectively and vice versa. A wider obtuse angle (> 90°) was observed between (E2 and E3) and between (En1 and En2) for PM and YR, respectively which specified negative association (Fig. 7A and B). This finding suggested that genotypes excelling in E2 and En1 would not maintain the same level of performance in E3 and En2, respectively, and vice versa.

**Fig. 8:**
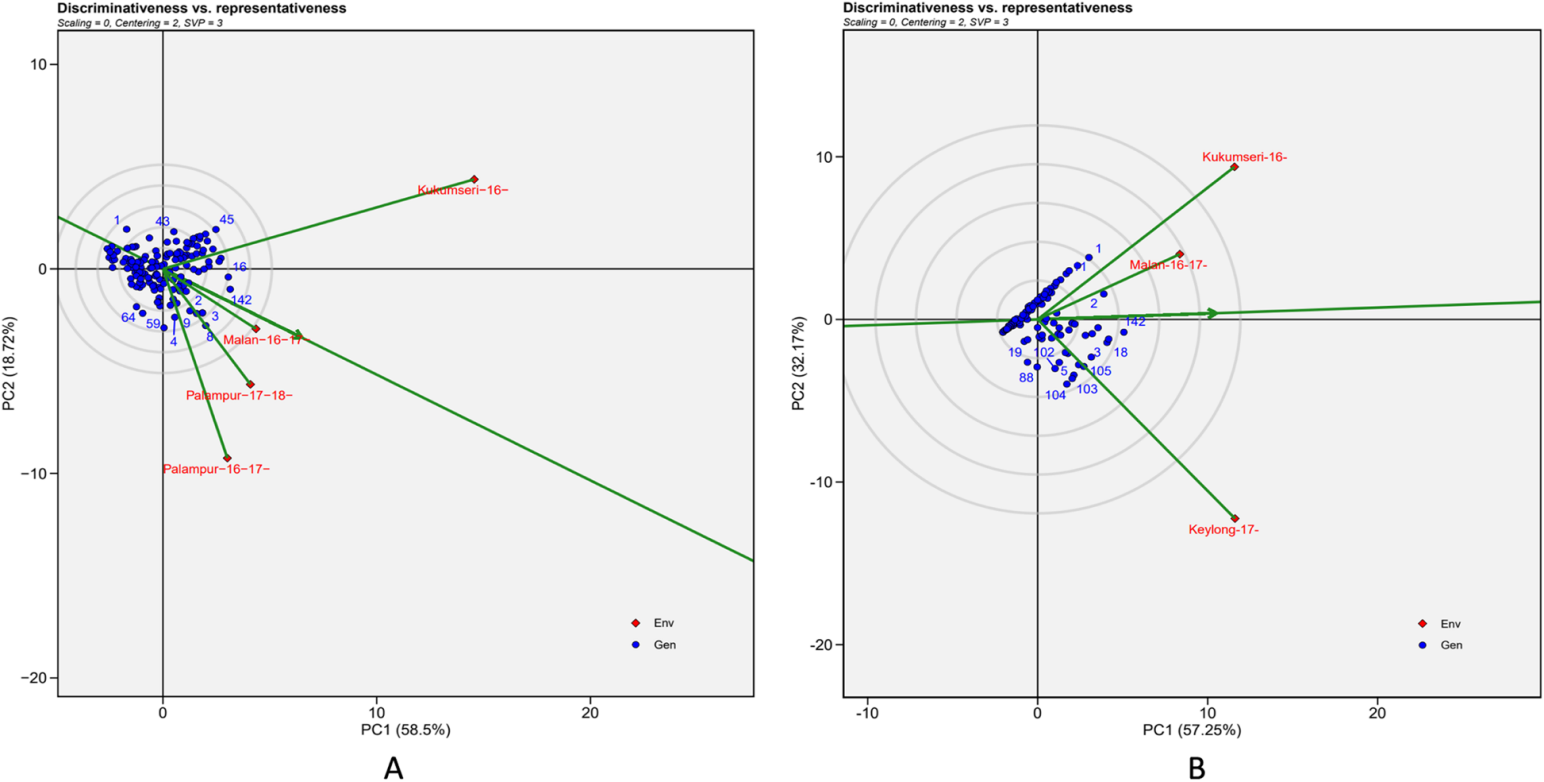
“Discrimitiveness vs representativeness” view of the GGE biplot of 142 wheat genotypes across 4 and 3 testing locations for PM and YR, respectively. There was no transformation of data (transform = 0), and data were centred by means of the environments (centring = 2). The biplot was based on symmetrically scaled (SVP = 3). **A** Powdery mildew (PM) {Malan-16–17- = E1 (Malan 2016–17); Kukumseri-16- = E2 (Kukumseri 2016); Palampur-16–17- = E3 (Palampur 2016–17); Palampur-17–18- = E4 (Palampur 2017–18) and **B**) Yellow rust (YR) {Kukumseri-16- = En1 (Kukumseri 2016); Keylong-17- = En2 (Keylong 2017); Malan-16–17- = En3 (Malan 2016–17)} Env = environment, Gen = genotype

Discriminativeness vs representativeness view of the biplot for test environments revealed that E2 and En1 had the longest vector length than other environments for PM and YR, respectively. This suggested that these environments had the greatest discrimination potential and the highest capability for genetic differentiation of genotypes. In contrast, E1 and En3, with the shortest vector length indicated that the least discriminatory environment for PM and YR, respectively. The representativeness of an environment is determined by the angle between the test environment vector and the “AEC abscissa”, a single-arrowed line passing through the biplot origin and representing the average of all environments (Fig. 8A and B). The E2 and En1 exhibited greater angle with the AEC abscissa, indicating lower representativeness due to their higher PM and YR disease pressure, respectively (Fig. 8A and B). Therefore, these environments are the most suitable for identifying the best disease-resistance genotypes. Hence, based on the highest discriminating ability, environments E2 and En1 may be recommended as the hot spots for screening against PM and YR, respectively.

### Stability, adaptability and performance of genotypes

AMMI-based stability parameters (ASV, IPCA 1, and IPCA 2) were computed based on the first two IPCAs to generate a well-balanced measure for evaluation of PM and YR severity. AMMI stability values (ASV) ranges from 0.12–6.78 and 0.37–11.47 for PM and YR, respectively. It was observed in the present study that, genotypes with higher ASV i.e. > 3 and > 7 for PM and YR disease, respectively revealed that these genotypes experience lower disease severity at some locations, whereas severity was higher in other genotypes, highlighting substantial location-based differences in disease response. The genotypes G-16, G-17, G-22, G-26, G-33, G-39, G-45, G-46, G-47, G-49, G-52, G-53, G-56, G-58, G-59, G-64, and G-127 exhibited an ASV greater than 3, indicating that PM disease severity varied significantly across different locations. Similarly, genotypes i.e. G-1, G-4, G-10, G-11, G-30, G-88, G-94, G-102, G-103, G-104 and G-105 showed ASV > 7 for YR, indicating that YR severity also varied significantly across different locations (Table S1).

The genotypes with ASV of close to 0 for PM and YR, experienced stable disease severity, either higher or lower in all the test locations. Genotypes G-13, G-62, G-7, G-81, G-112 and G-136 having ASV close to 0, showed stable PM disease severity (higher/lower), whereas genotypes G-51, G-125, G-23, G-106, G-83, G-92, G-84, G-90, G-131, G-82, G-68 and G-128, expressed stable YR disease severity (higher/lower) at all the locations (Table S1).

The AMMI 1 biplot clearly demonstrated that genotypes G-1, G-28, G-120, G-121, G-122, G-123, G-124, G-125, G-126 and G-128, positioned on the left side of the central axis recorded low level of PM disease severity (Fig. 4A). In contrast, genotypes G-2, G-3, G-8, G-9, G-16, G-22 and G-142 showed high level of PM disease severity. Genotypes G-7, G-12, G-13, G-16, G-17, G-20, G-23, G-28, G-33, G-55, G-60, G-61, G-68, G-69, G-75, G-83, G-84, G-86, G-87, G-89, G-90, G-92, G-125, G-128, G-129, G-130, G-131, G-132, G-133, G-134, G-135, G-136, G-137, G-138, G-139, G-140 and G-141, were positioned on the left side of the central axis and showed low levels of YR severity (Fig. 4B). Genotypes viz. G-1, G-2, G-3, G-18, G-96, G-97 and G-142 located at the right of the central axis, exhibited the highest YR severity.

Genotypes with IPCA-1 scores of zero or close to zero (either positive or negative) exhibited small interactions and had greater stability over the environments whereas, those with large IPCA-1 scores exhibited high interaction effect and exhibited more specific stability to specific environments [15, 42]. Genotypes G-45 and G-46 recorded the highest IPCA-1 score of 1.00 and 0.78, respectively followed by genotypes i.e. G-17, G-22, G-26, G-39, G-41, G-52, G-53, G-56, G-58 and G-64 with IPCA-1 score of > 0.6 (either positive or negative) for PM. Genotypes G-1, G-4, G-5, G-8, G-10, G-11, G-19, G-30, G-88, G-94, G-102, G-103, G-104 and G-105 recorded IPCA-1 score > 0.6 (either positive or negative) for YR. These observations inferred that the genotypes with higher IPCA-1 scores exhibited a stronger GEI, reflecting their specific adoption and stability in certain environments (Table-S1).

In AMMI 2 biplot, it was observed that some of the genotypes clustered together and were close to the origin for both PM and YR (Fig. 5A and B), suggesting that these genotypes exhibited a similar and high level of disease resistance across environments. Genotypes located close to the test environment in the biplot showed better adoption to that environment. Therefore, it is clear from the biplot that under test environments E1 and E2, genotypes G-138 and G-45 had consistently high level of PM severity. Furthermore, the biplot showed that many genotypes exhibited susceptibility to YR across all test environments, indicating that these environments provided conducive environment for disease expression for screening against YR.

The “which-won-where” biplot analysis effectively identified genotypes with specific adaptive abilities for particular environments or groups of environments [43]. In biplot, polygon was created by connecting the markers of the genotypes that were farthest from the biplot origin, thus encompassing all genotypes within it. Genotypes located at the convex hull positions exhibited both maximum and the minimum severity of PM and YR, making the greatest contribution to the GEI. On the other hand, genotypes inside the polygon were less responsive to GEI. The which-won-where biplot analysis also revealed that the genotypes at the left side of the convex hull showed less severity in all the environments, while those on the right side exhibited maximum disease severity across the environments. It was observed that genotypes G-1, G-28, G-120, G-121, G-122, G-123, G-124, G-125, G-126 and G-128 showed high level of resistance to PM. Six genotypes i.e. G-2, G-3, G-8, G-9, G-16, G-22 and susceptible check i.e. G-142 (Lehmi) showed high level of PM severity as was evident from their placement at the far-right outer boundary of biplot origin (Fig. 6A; Table 2). In case of YR, genotypes G-7, G-12, G-13, G-16, G-17, G-20, G-23, G-28, G-33, G-55, G-60, G-61, G-68, G-69, G-75, G-83, G-84, G-86, G-87, G-89, G-90, G-92, G-125, G-128, G-129, G-130, G-131, G-132, G-133, G-134, G-135, G-136, G-137, G-138, G-139, G-140 and G-141 showed low disease severity. Contrarily, genotypes i.e. G-1, G-2, G-3, G-18, G-96, G-97 and susceptible check G-142 (Lehmi) expressed high YR severity (Fig. 6B; Table 2).

**Table 2:** Information on the genotypes, their pedigree, origin of the test genotypes and *Yr*, *Lr* and *Pm* genes. Terminal powdery mildew (PM) and yellow rust (YR) severity (%) at adult plant stage, at CSKHPKV Palampur during winter (2016–17 and 2017–18), HAREC Kukumseri (summer 2016), Keylong (summer 2017) and RWRC Malan during winter (2016–17)

S. No	Genotypes	Origin	*Pm*, *Yr* and *Lr* genes	Infection type of powdery mildew at Seedling stage	Disease severity at adult plant stage against								
					**Powdery mildew (PM) at**					**Yellow rust (YR) at**			
					**Malan-16–17- (E1)**	**Kukumseri-16- (E2)**	**Palampur-16–17- (E3)**	**Palampur-17–18- (E4)**	**Mean**	**Kukumseri 2016 (En1)**	**Keylong 2017 (En2)**	**Malan-16–17 (En3)**	**Mean**
G-1	Morocco	PAU	-	2	0.00	20.00	0.00	0.00	5.00	90.00	0.00	80.00	56.67
G-2	Avocet-*Yra*	PAU	*Yra*	3	20.00	55.00	50.00	40.00	41.25	90.00	40.00	60.00	63.33
G-3	Avocet + *Yra*	PAU	*Yra*	2	20.00	55.00	50.00	40.00	41.25	70.00	80.00	40.00	63.33
G-4	*Yr1*/6*AOC	PAU	*Yr1,*	3	20.00	35.00	50.00	30.00	33.75	50.00	80.00	0.00	43.33
G-5	Siete Cerros T66	PAU	*Lr34, Lr13, Pm5b*	3	30.00	46.75	20.00	40.00	34.19	20.00	80.00	30.00	43.33
G-6	Tatara (*Yr 3,Yr 29* +)	PAU	*Yr3, Yr4, Yr9, Lr10, Lr13, Lr26, Lr27, Lr31*	3	40.00	32.00	15.00	40.00	31.75	20.00	0.00	20.00	13.33
G-7	*Yr5*/6*AOC	PAU	*Yr5*	3	30.00	43.75	25.00	40.00	34.69	5.00	0.00	10.00	5.00
G-8	*Yr6*/6*AOC	PAU	*Yr6*	3	40.00	50.00	45.00	50.00	46.25	80.00	0.00	40.00	40.00
G-9	*Yr7*/6*AOC	PAU	*Yr7*	3	40.00	46.25	40.00	40.00	41.56	60.00	0.00	40.00	33.33
G-10	*Yr8*/6*AOC	PAU	*Yr8*	3	20.00	24.00	30.00	40.00	28.50	85.00	0.00	40.00	41.67
G-11	*Yr9*/6*AOC	PAU	*Yr9*	3	40.00	40.00	20.00	30.00	32.50	85.00	0.00	60.00	48.33
G-12	*Yr10*/6*AOC	PAU	*Yr10*	3	30.00	35.00	30.00	40.00	33.75	2.00	0.00	0.00	0.67
G-13	*Yr15*/6*AOC	PAU	*Yr15*	4	30.00	42.50	20.00	40.00	33.13	0.00	0.00	0.00	0.00
G-14	*Yr17*/6*AOC	PAU	*Yr17*	3	20.00	37.50	5.00	30.00	23.13	30.00	0.00	20.00	16.67
G-15	*Yr18*/3*AOC	PAU	*Yr18*	3	30.00	43.75	40.00	40.00	38.44	40.00	0.00	5.00	15.00
G-16	*Yr24*/3*AOC	PAU	*Yr24*	3	30.00	77.50	30.00	40.00	44.38	20.00	0.00	0.00	6.67
G-17	*Yr26*/3*AOC	PAU	*Yr26*	3	30.00	70.00	5.00	40.00	36.25	10.00	0.00	0.00	3.33
G-18	*Yr27*/6*AOC	PAU	*Yr27*	2	40.00	27.75	25.00	40.00	33.19	80.00	80.00	30.00	63.33
G-19	*YrSP*/6*AOC	PAU	*YrSP*	3	30.00	47.50	40.00	0.00	29.38	2.00	40.00	0.00	14.00
G-20	Pavon F 76 (*Yr 6, Yr 7, Yr 29, Yr 30* +)	PAU	*Lr1, Lr2b, Lr10, Lr13, Lr14a, Lr22a/b, Lr27, Lr46* + *, Yr2, Yr6, Yr7, Yr8, Yr29, Yr30, Lr46/Yr29/Sr58/Pm39, Sr2/Yr30, Lr46/Yr29/Sr58*	3	30.00	32.50	5.00	30.00	24.38	2.00	0.00	20.00	7.33
G-21	Seri M 82 (*Yr 2, Yr 9, Yr 29, Yr 30* +)	PAU	*Yr 2, Yr 9, Yr 29, Yr 30* +	3	20.00	32.50	25.00	0.00	19.38	15.00	30.00	30.00	25.00
G-22	Opata M 85 (*Yr 27, Yr 18, Yr 30* +)	PAU	*Lr10, Lr13, Lr14b, Lr30, Lr27, Lr31, Lr34* + *, Yr18, Yr27, Yr30*	3	40.00	72.50	10.00	40.00	40.63	30.00	0.00	20.00	16.67
G-23	Super Kauz (*Yr 9, Yr 27, Yr 18, Yr30* +)	PAU	*Yr 9, Yr 27, Yr 18, Yr30* +	3	30.00	51.25	7.00	30.00	29.56	10.00	0.00	10.00	6.67
G-24	*Yr*cv/6*AOC	PAU	*-*	3	30.00	51.25	7.00	30.00	29.56	40.00	40.00	5.00	28.33
G-25	AOC-Yr*3/3/Altar 84/Ae.sq//Opata	PAU	*-*	3	20.00	16.67	3.00	30.00	17.42	25.00	20.00	20.00	21.67
G-26	AOC-YR*3//Lalbmon O1 *4/PVN	PAU	*-*	3	20.00	63.75	2.00	30.00	28.94	40.00	40.00	20.00	33.33
G-27	AOC-Yr*3/Pastor	PAU	*Yr31, Yr29, Yr30, Yr31, Pm4b*	2	20.00	43.75	0.00	40.00	25.94	30.00	30.00	3.00	21.00
G-28	Pollmer/CTY88.547	PAU	*-*	2	0.00	0.00	0.00	20.00	5.00	0.00	0.00	0.00	0.00
G-29	Thatcher	PAU	*-*	3	40.00	38.75	0.00	10.00	22.19	30.00	60.00	30.00	40.00
G-30	NIL-Thatcher-*Lr1*-CTR	PAU	*Lr1*	3	40.00	47.50	5.00	25.00	29.38	15.00	60.00	30.00	35.00
G-31	NIL-Thatcher-*Lr2a*-WST	PAU	*Lr2a*	3	30.00	52.50	5.00	25.00	28.13	60.00	60.00	30.00	50.00
G-32	NIL-Thatcher-*Lr2b*-Carina	PAU	*Lr1, Lr2b, Lr2c*	4	30.00	47.50	10.00	25.00	28.13	40.00	35.00	30.00	35.00
G-33	NIL-Thatcher-*Lr2c*-Loros	PAU	*Lr2c, Lr2 d*	3	40.00	57.50	0.00	20.00	29.38	2.00	0.00	20.00	7.33
G-34	NIL-Thatcher-*Lr3*-Democrat	PAU	*Lr3, Lr3a*	3	40.00	47.50	10.00	20.00	29.38	2.00	20.00	20.00	14.00
G-35	IL-Thatcher-*Lr3*KA-AIV	PAU	*Lr3ka*	3	30.00	41.25	15.00	25.00	27.81	50.00	0.00	40.00	30.00
G-36	NIL-Thatcher-*Lr3*BG-Bage	PAU	*Lr3bg*	3	40.00	55.00	0.00	25.00	30.00	30.00	0.00	40.00	23.33
G-37	NIL-Thatcher-*Lr9*-Tranfer	PAU	*Lr9*	3	40.00	52.50	5.00	30.00	31.88	20.00	0.00	20.00	13.33
G-38	Hussar	PAU	*Lr11*	3	40.00	56.25	5.00	30.00	32.81	25.00	0.00	30.00	18.33
G-39	NIL-Thatcher-*Lr12*-EX	PAU	*Lr12*	2	30.00	65.00	10.00	15.00	30.00	50.00	0.00	30.00	26.67
G-40	Manitou	PAU	*Lr13, Lr22b, Lr23, Lr34*	3	30.00	46.25	5.00	25.00	26.56	50.00	0.00	20.00	23.33
G-41	RL 6006	PAU	*Lr14b*	3	40.00	66.25	0.00	30.00	34.06	40.00	0.00	30.00	23.33
G-42	NIL-Thatcher-*Lr16*-EX	PAU	*Lr16*	3	30.00	60.00	4.00	30.00	31.00	40.00	0.00	20.00	20.00
G-43	NIL-Thatcher-*Lr19*-TR	PAU	*Lr19*	3	20.00	48.75	0.00	15.00	20.94	60.00	0.00	40.00	33.33
G-44	NIL-Thatcher-*Lr21*-RL5406	PAU	*Lr21, Lr42*	3	20.00	57.50	10.00	20.00	26.88	50.00	0.00	30.00	26.67
G-45	NIL-Thatcher-*Lr22a*-RL5404	PAU	*Lr22a, Lr42*	3	20.00	80.00	10.00	20.00	32.50	40.00	0.00	40.00	26.67
G-46	NIL-Thatcher-*Lr23*-LEE310	PAU	*Lr23*	3	30.00	68.75	2.00	25.00	31.44	60.00	0.00	40.00	33.33
G-47	NIL-Thatcher-*Lr24*-Agent	PAU	*Lr1, Lr10, Lr24*	3	20.00	68.75	30.00	25.00	35.94	50.00	0.00	30.00	26.67
G-48	Transec (Awned)-*Lr25*	PAU	*Lr1, Lr2a, Lr2c, Lr25, Pm7*	3	40.00	45.00	2.00	25.00	28.00	60.00	25.00	10.00	31.67
G-49	NIL-Thatcher-*Lr26*-ST-1–25	PAU	*Lr26*	3	20.00	65.00	15.00	30.00	32.50	40.00	40.00	20.00	33.33
G-50	Gatcher	PAU	*Yr7, Lr10, Lr12, Lr14a, Lr27, Lr31*	3	20.00	62.50	20.00	40.00	35.63	20.00	0.00	30.00	16.67
G-51	NIL-Thatcher-*Lr29*-CS7 AG11	PAU	*Lr29*	3	10.00	50.00	5.00	25.00	22.50	20.00	5.00	20.00	15.00
G-52	NIL-Thatcher-*Lr30*-Tzio	PAU	*Lr30*	3	20.00	67.50	20.00	20.00	31.88	15.00	0.00	30.00	15.00
G-53	NIL-Thatcher-*Lr32*-Ae.ta	PAU	*Lr32*	3	30.00	65.00	5.00	20.00	30.00	60.00	0.00	30.00	30.00
G-54	NIL-Thatcher-*Lr33*-PI58548	PAU	*Yr18, Lr1, Lr2, Lr33, Lr34, Lr44*	3	20.00	44.25	40.00	15.00	29.81	5.00	0.00	30.00	11.67
G-55	NIL-Thatcher-*Lr34*-PI58548	PAU	*Yr18, Lr1, Lr2, Lr33, Lr34, Lr44*	3	20.00	40.00	10.00	20.00	22.50	10.00	0.00	0.50	3.50
G-56	NIL-Manitou-*Lr36*-T.SP2-9	PAU	*Lr36*	3	20.00	70.00	30.00	20.00	35.00	40.00	0.00	20.00	20.00
G-57	NIL-Thatcher-*Lr37*-VPM	PAU	*Lr37*	3	20.00	30.50	25.00	20.00	23.88	20.00	0.00	20.00	13.33
G-58	NIL-Thatcher-*Lrb*-Carina	PAU	*Lr1, Lr2b, Lr2c, LrB*	3	20.00	75.00	20.00	40.00	38.75	10.00	0.00	30.00	13.33
G-59	WL 711	PAU	*Yr2, Lr13, Lr14a*	3	20.00	22.50	40.00	50.00	33.13	25.00	0.00	20.00	15.00
G-60	DW7276	PAU	*-*	3	20.00	35.00	30.00	40.00	31.25	0.00	0.00	0.00	0.00
G-61	Iumillo	PAU	*-*	3	20.00	23.75	7.00	30.00	20.19	10.00	0.00	0.00	3.33
G-62	Local Red	PAU	*Lr14a, Lr18*	3	20.00	20.25	7.00	0.00	11.81	70.00	0.00	40.00	36.67
G-63	*Lr41*/6*TC	PAU	*Lr41*	4	20.00	60.00	30.00	25.00	33.75	40.00	0.00	40.00	26.67
G-64	TC*6/T.spelta 783	PAU	*-*	3	30.00	10.00	35.00	25.00	25.00	40.00	5.00	30.00	25.00
G-65	TC/4 *ST-1	PAU	*-*	3	30.00	6.75	10.00	20.00	16.69	15.00	5.00	30.00	16.67
G-66	Pavon + *Lr47*	PAU	*Lr47*	3	20.00	57.50	30.00	20.00	31.88	50.00	5.00	30.00	28.33
G-67	C 78.5	PAU	-	3	20.00	41.25	7.00	25.00	23.31	40.00	0.00	20.00	20.00
G-68	HP 348	CSKHPKV	-	2	15.00	50.00	20.00	20.00	26.25	15.00	0.00	0.00	5.00
G-69	HPW 314	CSKHPKV	-	3	15.00	15.25	15.00	30.00	18.81	2.00	0.00	0.00	0.67
G-70	JAL95.4.3/3/Kachu # 1/Kiritati//Kachu	CIMMYT	-	3	5.00	26.25	10.00	20.00	15.31	40.00	0.00	30.00	23.33
G-71	JAL95.4.3/3/Kachu # 1/Kiritati//Kachu	CIMMYT	-	2	15.00	52.50	20.00	30.00	29.38	40.00	0.00	30.00	23.33
G-72	JAL95.4.3/3/Kachu # 1/Kiritati//Kachu	CIMMYT	-	3	10.00	33.75	10.00	0.00	13.44	30.00	0.00	20.00	16.67
G-73	JAL95.4.3/3/Kachu # 1/Kiritati//Kachu	CIMMYT	-	3	20.00	43.75	10.00	30.00	25.94	40.00	0.00	10.00	16.67
G-74	JAL95.4.3/3/Kachu # 1/Kiritati//Kachu	CIMMYT	-	3	20.00	35.00	20.00	30.00	26.25	30.00	0.00	20.00	16.67
G-75	JAL95.4.3/3/Kachu # 1/Kiritati//Kachu	CIMMYT	-	3	20.00	13.75	20.00	25.00	19.69	10.00	0.00	0.00	3.33
G-76	JAL95.4.3/3/Kachu # 1/Kiritati//Kachu	CIMMYT	-	3	30.00	22.50	20.00	40.00	28.13	10.00	0.00	20.00	10.00
G-77	JAL95.4.3/3/Kachu # 1/Kiritati//Kachu	CIMMYT	-	3	40.00	23.00	15.00	30.00	27.00	10.00	0.00	20.00	10.00
G-78	JAL95.4.3/3/Kachu # 1/Kiritati//Kachu	CIMMYT	-	3	30.00	14.00	15.00	25.00	21.00	1.00	0.00	30.00	10.33
G-79	JAL95.4.3/3/Kachu # 1/Kiritati//Kachu	CIMMYT	-	3	40.00	7.00	10.00	25.00	20.50	40.00	0.00	40.00	26.67
G-80	JAL95.4.3/3/Kachu # 1/Kiritati//Kachu	CIMMYT	-	3	40.00	35.00	15.00	30.00	30.00	10.00	0.00	40.00	16.67
G-81	JAL95.4.3/3/Kachu # 1/Kiritati//Kachu	CIMMYT	-	3	20.00	36.25	10.00	40.00	26.56	10.00	5.00	20.00	11.67
G-82	JAL95.4.3/3/Kachu # 1/Kiritati//Kachu	CIMMYT	-	3	20.00	8.00	15.00	25.00	17.00	20.00	5.00	5.00	10.00
G-83	JAL95.4.3/3/Kachu # 1/Kiritati//Kachu	CIMMYT	-	3	40.00	22.75	10.00	40.00	28.19	20.00	0.00	5.00	8.33
G-84	JAL95.4.3/3/Kachu # 1/Kiritati//Kachu	CIMMYT	-	3	30.00	24.25	10.00	30.00	23.56	10.00	0.00	5.00	5.00
G-85	JAL95.4.3/3/Kachu # 1/Kiritati//Kachu	CIMMYT	-	3	20.00	32.50	7.00	15.00	18.63	10.00	0.00	20.00	10.00
G-86	JAL95.4.3/3/Kachu # 1/Kiritati//Kachu	CIMMYT	-	3	40.00	20.25	15.00	25.00	25.06	0.00	0.00	0.00	0.00
G-87	JAL95.4.3/3/Kachu # 1/Kiritati//Kachu	CIMMYT	-	3	20.00	18.00	2.00	25.00	16.25	3.00	0.00	20.00	7.67
G-88	JAL95.4.3/3/Kachu # 1/Kiritati//Kachu	CIMMYT	-	3	30.00	20.25	5.00	15.00	17.56	5.00	50.00	5.00	20.00
G-89	JAL95.4.3/3/Kachu # 1/Kiritati//Kachu	CIMMYT	-	3	20.00	15.50	15.00	20.00	17.63	10.00	0.00	1.00	3.67
G-90	JAL95.4.3/3/Kachu # 1/Kiritati//Kachu	CIMMYT	-	3	30.00	29.25	0.00	25.00	21.06	10.00	0.00	5.00	5.00
G-91	JAL95.4.3/3/Kachu # 1/Kiritati//Kachu	CIMMYT	-	3	30.00	30.00	5.00	30.00	23.75	10.00	0.00	30.00	13.33
G-92	IG 41514/5/Seri.1B//Kauz/Hevo/3/Amad*2/4/Kiritati/6/Fret2 *2/4/SNI/Trap#1/3/Kauz*2/…	CIMMYT	*Yr6, Yr7, Yr9, Yr18, Yr27, Lr1, Lr3a, Lr10, Lr13, Lr26, Lr34,*	3	30.00	43.75	2.00	30.00	26.44	10.00	0.00	5.00	5.00
G-93	CPI18/Gediz/3/Goo//ALB/CRA/4/*AE. squarrosa* (494)/6/Kauz//Altar 84/…	CIMMYT	*Yr3, Yr6, Yr7, Yr9, Yr18, Yr27, Lr13, Lr23, Lr26*	4	20.00	55.00	2.00	30.00	26.75	50.00	0.00	20.00	23.33
G-94	PHSL 10	IIWBR	-	3	20.00	21.75	20.00	25.00	21.69	30.00	80.00	40.00	50.00
G-95	PHSL 11	IIWBR	-	3	30.00	13.00	15.00	10.00	17.00	40.00	40.00	60.00	46.67
G-96	AKW 4739	IIWBR	-	3	20.00	10.75	5.00	15.00	12.69	60.00	60.00	60.00	60.00
G-97	GW 2010–272	IIWBR	-	3	20.00	19.25	10.00	15.00	16.06	50.00	60.00	60.00	56.67
G-98	GW 2010–281	IIWBR	-	3	30.00	18.75	15.00	15.00	19.69	30.00	40.00	60.00	43.33
G-99	GW 2010–281	IIWBR	-	3	30.00	10.00	3.00	20.00	15.75	50.00	40.00	40.00	43.33
G-100	GW 2010–288	IIWBR	-	3	30.00	16.25	10.00	20.00	19.06	50.00	40.00	40.00	43.33
G-101	VW 20145	IIWBR	-	3	20.00	9.50	5.00	10.00	11.13	30.00	40.00	40.00	36.67
G-102	VW 20167	IIWBR	-	3	20.00	5.75	0.00	20.00	11.44	0.50	60.00	40.00	33.50
G-103	VW 20168	IIWBR	-	3	20.00	16.25	5.00	20.00	15.31	10.00	80.00	40.00	43.33
G-104	EIGSN 2013–16	IIWBR	-	2	20.00	28.75	20.00	20.00	22.19	0.75	80.00	40.00	40.25
G-105	EIGSN 2013–8	IIWBR	-	3	20.00	15.00	20.00	20.00	18.75	50.00	80.00	30.00	53.33
G-106	EGSN 2013–13	IIWBR	-	3	20.00	22.50	20.00	20.00	20.63	30.00	20.00	30.00	26.67
G-107	NW 5054	IIWBR	*Lr23* +, *Sr7b* +, *Yr2* +	3	20.00	17.50	0.00	10.00	11.88	40.00	0.00	30.00	23.33
G-108	EIGSN 2013–36	IIWBR	-	3	20.00	10.00	0.00	30.00	15.00	40.00	0.00	60.00	33.33
G-109	EIGSN 2013–55	IIWBR	-	3	30.00	10.00	0.00	35.00	18.75	20.00	0.00	40.00	20.00
G-110	AKAW 4731	IIWBR	-	3	20.00	13.75	15.00	15.00	15.94	10.00	0.00	40.00	16.67
G-111	GW 281	IIWBR	-	3	10.00	15.00	15.00	10.00	12.50	20.00	0.00	40.00	20.00
G-112	VAS 320	IIWBR	-	3	10.00	17.50	0.00	15.00	10.63	40.00	40.00	60.00	46.67
G-113	ALY 1090	IIWBR	-	2	5.00	15.00	7.00	20.00	11.75	0.55	20.00	30.00	16.85
G-114	HE 1584	IIWBR	-	2	10.00	13.00	0.00	30.00	13.25	50.00	0.00	40.00	30.00
G-115	MP 1259	IIWBR	-	2	15.00	15.00	7.00	30.00	16.75	40.00	0.00	40.00	26.67
G-116	NP 3288	IIWBR	-	2	10.00	10.00	10.00	20.00	12.50	30.00	0.00	20.00	16.67
G-117	NIAW 34	IIWBR	*Sr8a* +, *Sr11*, *Lr13*, *Lr26*, *Lr34*, *Yr18*	4	15.00	17.50	15.00	20.00	16.88	60.00	0.00	20.00	26.67
G-118	NW 5013	IIWBR	-	3	5.00	8.75	10.00	0.00	5.94	50.00	0.00	30.00	26.67
G-119	PHS 1101	IIWBR	-	3	10.00	15.00	15.00	25.00	16.25	20.00	0.00	20.00	13.33
G-120	Axminister	IIWBR	*Pm1a*	3	0.00	3.50	10.00	10.00	5.88	50.00	30.00	10.00	30.00
G-121	Ulka	IIWBR	*Pm2*	2	0.00	2.50	15.00	10.00	6.88	70.00	0.00	30.00	33.33
G-122	Asosan	IIWBR	*Pm3a*	3	0.00	5.00	0.00	10.00	3.75	30.00	0.00	30.00	20.00
G-123	Chul	IIWBR	*Pm3b*	2	0.00	1.25	0.00	10.00	2.81	40.00	20.00	20.00	26.67
G-124	Sonora	IIWBR	*Pm3c*	2	0.00	3.00	0.00	25.00	7.00	30.00	30.00	10.00	23.33
G-125	Syros	IIWBR	*-*	3	0.00	3.50	0.00	20.00	5.88	10.00	0.00	10.00	6.67
G-126	Khapli	IIWBR	*Pm4a*	3	0.00	4.25	10.00	0.00	3.56	45.00	0.00	20.00	21.67
G-127	Hope	IIWBR	*Pm5a*	4	10.00	11.25	40.00	20.00	20.31	40.00	60.00	10.00	36.67
G-128	Talent	IIWBR	*Pm5* + *?*	3	0.00	15.00	0.00	20.00	8.75	20.00	0.00	0.75	6.92
G-129	Mercato//Parus/Pastor	IIWBR	*Pm4b, Lr14a, Yr29, Yr30, Yr31, Lr37*	2	40.00	23.75	5.00	15.00	20.94	3.00	0.00	0.00	1.00
G-130	Quaiu #1*2/Munal #1	IIWBR	*Yr29, Yr30* +	3	40.00	20.50	25.00	30.00	28.88	5.00	0.00	10.00	5.00
G-131	Quaiu #1*2/Munal #1	IIWBR	*Yr29, Yr30* +	3	40.00	36.25	15.00	45.00	34.06	10.00	0.00	5.00	5.00
G-132	Urbina S2007*2/3/Guam92/Kauz//Zhengyou 6	IIWBR	*Yr6, Yr7, Yr9, Yr18, Yr27, Lr13, Lr26*	3	20.00	21.75	15.00	43.00	24.94	2.00	0.00	5.00	2.33
G-133	PRL/2*Pastor//Kachu	IIWBR	*Yr29, Yr30, Yr31, Pm4b*	2	30.00	14.50	15.00	18.00	19.38	5.00	0.00	10.00	5.00
G-134	Sokoll/3/Pastor//HXL7573/2*BAU*2/4/NAVJ07	IIWBR	*Pm4b, Yr9, Lr26, Yr29, Yr30, Yr31*	2	15.00	15.50	10.00	20.00	15.13	3.00	0.00	5.00	2.67
G-135	Babax/*Lr42*//Babax*2/3/Pavon7S3, + *Lr47*/4/Rolf07/Yanac//Tacupeto F2001/Brambling	IIWBR	*Lr14a, Lr23, Lr27, Lr31, Lr34, Lr42, Lr47*	3	35.00	18.25	10.00	25.00	22.06	2.00	0.00	10.00	4.00
G-136	Fret2/Kukuna//Fret2/3/Yanac/4/Fret2/Kiritati*2/5/Whear//2*PRL/2*Pastor	IIWBR	*Pm8, Yr29, Yr30, Yr31, Lr34* +	3	20.00	30.00	15.00	20.00	21.25	5.00	0.00	5.00	3.33
G-137	ND643/2*Wbll1//Villa Juarez F2009	IIWBR	*-*	3	40.00	23.75	7.00	28.00	24.69	10.00	0.00	0.00	3.33
G-138	Mutus*2//TAM200/Turaco	IIWBR	*Pm17, Lr3a, Lr24*	2	45.00	17.75	10.00	30.00	25.69	2.00	0.00	5.00	2.33
G-139	Mutus*2//Haril #1	IIWBR	-	3	30.00	24.25	10.00	20.00	21.06	2.00	0.00	5.00	2.33
G-140	Mutus*2/Haril #1	IIWBR	-	3	30.00	28.75	15.00	35.00	27.19	5.00	0.00	5.00	3.33
G-141	Quaiu/Becard//Becard	IIWBR	-	3	40.00	20.00	25.00	30.00	28.75	3.00	0.00	5.00	2.67
G-142	Check Lehmi			4	40.00	75.00	35.00	40.00	47.50	70.00	80.00	80.00	76.67
	Mean				23.98	32.92	13.66	25.06	23.90	28.84	13.66	23.70	22.06

- data not available; PAU= Punjab Agricultural University, Ludhiana; CSKHPV= Chaudhary Sarwan Kumar Himachal Pradesh Krishi Vishvavidyalaya, Palampur; CIMMYT= CIMMYT, Mexico; IIWBR= Indian Institute of Wheat and Barley Research, Karnal.

# Information on *Pm*, *Yr* and *Lr* resistance genes in test varieties was collected from CIMMYT Genetic Resources Information System for Wheat and Triticale (GRIS) Accessions List (wheatpedigree.net)

*Malan-16–17- = Malan (2016-17); Kukumseri-16- = Kukumseri (2016); Palampur-16–17- = Palampur (2016-17); Palampur-17–18- = Palampur (2017-18) Keylong-17- = Keylong (2017)

### Resistance to powdery mildew (PM) at seedling stage

None of the genotype was free from PM. Twenty genotypes i.e. G-1, G-3, G-18, G-27, G-28, G-39, G-68, G-71, G-104, G-113, G-114, G-115, G-116, G-121, G-123, G-124, G-129, G-133, G-134 and G-138 with IT = ‘2’ were moderately resistant (Table 2).

### Slow mildewing resistance

Genotypes viz., G-21, G-25, G-62, G-65, G-70, G-72, G-96, G-99, G-102, G-107, G-108, G-109, G-112, G-113, G-114, G-115, G-116, G-117, G-118, G-119, G-120, G-121, G-122, G-123, G-124, G-125, G-126, G-128, G-133 and G-134 showed susceptible reaction at seedling stage but exhibited resistance at adult plant stage with mean terminal disease severity (TDS), AUDPC, rAUDPC and infection rate of ≤ 20%, ≤ 750, 39–50 and 0.01–0.06 units/day. TDS, AUDPC, rAUDPC and r in susceptible cultivar Lehmi was 47.50%, 1339.75, 100 and 0.05, respectively, whereas, TDS, AUDPC, rAUDPC and r varied between 20.31–46.25%, 754.06–1240.94, 50.20–97.54 and 0.01–0.06 units/day, respectively. So, therefore based on these metrics, these genotypes were classified as slow mildewing genotypes (Table 3).

**Table 3:** Area under disease progress curve (AUDPC), relative area under disease progress curve (rAUDPC) and infection rate ‘r’ of powdery mildew in wheat varieties at CSKHPKV Palampur during winter 2016–17, HAREC Kukumseri during summer 2016 and RWRC Malan during winter 2016–17

		**AUDPC**				**Mean**	**rAUDPC**				**Mean**	**Infection rate (r)**				**Mean**
**S. No**	**Genotypes**	**Malan**	**Kukum** **seri**	**Palam** **pur-17-**	**Palam** **pur-18-**		**Malan**	**Kukum seri**	**Palam** **pur-17-**	**Palam pur-18-**		**Malan**	**Kuku** **mseri**	**Palam** **pur- 17-**	**Palam** **pur-18-**	
G-1	Morocco	0.00	622.50	0.00	0.00	155.63	0.00	27.21	0.00	0.00	6.80	0.00	0.05	0.00	0.00	0.01
G-2	Avocet-*Yra*	913.50	1818.75	1123.50	750.00	1151.44	50.12	79.51	95.73	98.11	80.86	0.05	0.04	0.11	0.02	0.06
G-3	Avocet + *Yra*	832.50	1950.00	843.50	862.50	1122.13	45.68	85.25	46.95	27.78	51.41	0.03	0.04	0.11	0.03	0.05
G-4	*Yr1*/6*AOC	1102.50	971.25	962.50	637.50	918.44	60.49	42.46	67.68	94.44	91.27	0.03	0.04	0.07	0.02	0.04
G-5	Siete Cerros T66	967.50	1569.38	493.50	750.00	945.09	53.09	68.61	85.98	111.11	79.69	0.05	0.04	0.03	0.02	0.03
G-6	Tatara (*Yr 3,Yr 29* +)	1336.50	926.25	287.00	825.00	843.69	73.33	40.49	50.00	122.22	71.51	0.05	0.05	0.04	0.04	0.04
G-7	*Yr5*/6*AOC	931.50	1224.38	525.00	637.50	829.59	51.11	53.52	91.46	94.44	72.64	0.04	0.05	0.04	0.06	0.05
G-8	*Yr6*/6*AOC	1215.00	1650.00	805.00	750.00	1105.00	66.67	72.13	140.24	111.11	97.54	0.04	0.04	0.07	0.05	0.05
G-9	*Yr7*/6*AOC	877.50	1464.38	724.50	900.00	991.59	48.15	64.02	126.22	133.33	92.93	0.06	0.05	0.05	0.02	0.04
G-10	*Yr8*/6*AOC	945.00	783.75	612.50	675.00	754.06	51.85	34.26	106.71	100.00	73.21	0.02	0.04	0.09	0.04	0.05
G-11	*Yr9*/6*AOC	967.50	1680.00	423.50	600.00	917.75	53.09	73.44	73.78	88.89	72.30	0.06	0.02	0.04	0.03	0.04
G-12	*Yr10*/6*AOC	778.50	1293.75	542.50	637.50	813.06	42.72	56.56	94.51	94.44	72.06	0.06	0.02	0.09	0.06	0.06
G-13	*Yr15*/6*AOC	1170.00	1556.25	241.50	637.50	901.31	64.20	68.03	42.07	94.44	67.19	0.03	0.03	0.08	0.06	0.05
G-14	*Yr17*/6*AOC	823.50	1556.25	56.00	532.50	742.06	45.19	68.03	9.76	78.89	50.47	0.05	0.02	0.04	0.08	0.05
G-15	*Yr18*/3*AOC	1048.50	1603.13	647.50	862.50	1040.41	57.53	70.08	112.80	127.78	92.05	0.06	0.03	0.10	0.06	0.06
G-16	*Yr24*/3*AOC	1170.00	2231.25	402.50	637.50	1110.31	64.20	97.54	70.12	94.44	81.58	0.03	0.07	0.09	0.06	0.06
G-17	*Yr26*/3*AOC	1192.50	2231.25	56.00	787.50	1066.81	65.43	97.54	9.76	116.67	72.35	0.05	0.05	0.04	0.06	0.05
G-18	*Yr27*/6*AOC	1201.50	1014.38	259.00	787.50	815.59	65.93	44.34	45.12	116.67	68.01	0.05	0.03	0.08	0.06	0.05
G-19	*YrSP*/6*AOC	1228.50	1410.00	612.50	0.00	812.75	67.41	61.64	106.71	0.00	58.94	0.06	0.06	0.10	0.00	0.05
G-20	Pavon F 76 (*Yr 6, Yr 7, Yr 29, Yr 30* +)	967.50	1087.50	56.00	937.50	762.13	53.09	47.54	9.76	138.89	62.32	0.05	0.03	0.04	0.02	0.03
G-21	Seri M 82 (*Yr 2, Yr 9, Yr 29, Yr 30* +)	742.50	1207.50	427.00	0.00	594.25	40.74	52.79	74.39	0.00	41.98	0.03	0.03	0.08	0.00	0.04
G-22	Opata M 85 (*Yr 27, Yr 18, Yr 30* +)	1305.00	2287.50	262.50	607.50	1115.63	71.60	100.00	45.73	90.00	76.83	0.04	0.05	0.06	0.09	0.06
G-23	Super Kauz (*Yr 9, Yr 27, Yr 18, Yr30* +)	1192.50	1509.38	245.00	712.50	914.84	65.43	65.98	42.68	105.56	69.91	0.05	0.05	0.01	0.05	0.04
G-24	*Yr*cv/6*AOC	868.50	1659.38	245.00	562.50	833.84	47.65	72.54	42.68	83.33	61.55	0.06	0.04	0.01	0.05	0.04
G-25	AOC-Yr*3/3/Altar 84/Ae.sq//Opata	598.50	567.50	49.00	750.00	491.25	32.84	24.81	8.54	111.11	44.32	0.05	0.03	0.03	0.03	0.03
G-26	AOC-YR*3//Lalbmon O1 *4/PVN	787.50	1828.13	45.50	750.00	852.78	43.21	79.92	7.93	111.11	60.54	0.03	0.06	0.02	0.03	0.04
G-27	AOC-Yr*3/Pastor	832.50	1734.38	0.00	825.00	847.97	45.68	75.82	0.00	122.22	60.93	0.03	0.03	0.00	0.04	0.02
G-28	Pollmer/CTY88.547	0.00	0.00	0.00	172.50	43.13	0.00	0.00	0.00	25.56	6.39	0.00	0.00	0.00	0.07	0.02
G-29	Thatcher	1755.00	1321.88	0.00	678.75	938.91	96.30	57.79	0.00	100.56	63.66	0.04	0.04	0.00	0.07	0.04
G-30	NIL-Thatcher-*Lr1*-CTR	1957.50	1687.50	56.00	600.00	1075.25	107.41	73.77	9.76	88.89	69.96	0.03	0.03	0.04	0.03	0.03
G-31	NIL-Thatcher-*Lr2a*-WST	1710.00	1725.00	56.00	600.00	1022.75	93.83	75.41	9.76	88.89	66.97	0.03	0.04	0.04	0.03	0.04
G-32	NIL-Thatcher-*Lr2b*-Carina	1620.00	1425.00	269.50	600.00	978.63	88.89	62.30	46.95	88.89	71.76	0.03	0.05	0.03	0.03	0.04
G-33	NIL-Thatcher-*Lr2c*-Loros	1485.00	1875.00	0.00	562.50	980.63	81.48	81.97	0.00	83.33	61.70	0.04	0.04	0.00	0.05	0.03
G-34	NIL-Thatcher-*Lr3*-Democrat	1305.00	1575.00	234.50	562.50	919.25	71.60	68.85	40.85	83.33	66.16	0.04	0.04	0.03	0.05	0.04
G-35	IL-Thatcher-*Lr3*KA-AIV	1462.50	1509.38	364.00	600.00	983.97	80.25	65.98	63.41	88.89	74.63	0.02	0.03	0.03	0.03	0.03
G-36	NIL-Thatcher-*Lr3*BG-Bage	1147.50	1931.25	0.00	600.00	919.69	62.96	84.43	0.00	88.89	59.07	0.06	0.04	0.00	0.05	0.03
G-37	NIL-Thatcher-*Lr9*-Tranfer	1093.50	1762.50	84.00	637.50	894.38	60.00	77.05	14.63	94.44	61.53	0.07	0.03	0.04	0.03	0.04
G-38	Hussar	1440.00	1903.13	84.00	750.00	1044.28	79.01	83.20	14.63	111.11	71.99	0.02	0.04	0.04	0.02	0.03
G-39	NIL-Thatcher-*Lr12*-EX	1620.00	2475.00	136.50	675.00	1226.63	88.89	108.20	23.78	100.00	80.22	0.03	0.03	0.06	0.03	0.04
G-40	Manitou	1327.50	1884.38	84.00	712.50	1002.09	72.84	82.38	14.63	105.56	68.85	0.05	0.02	0.04	0.03	0.03
G-41	RL 6006	1485.00	2503.13	0.00	637.50	1156.41	81.48	109.43	0.00	94.44	71.34	0.04	0.03	0.00	0.08	0.04
G-42	NIL-Thatcher-*Lr16*-EX	1530.00	2306.25	52.50	675.00	1140.94	83.95	100.82	9.15	100.00	73.48	0.03	0.03	0.03	0.06	0.04
G-43	NIL-Thatcher-*Lr19*-TR	913.50	1753.13	0.00	416.25	770.72	50.12	76.64	0.00	61.67	47.11	0.05	0.03	0.00	0.03	0.03
G-44	NIL-Thatcher-*Lr21*-RL5406	1485.00	1968.75	136.50	637.50	1056.94	81.48	86.07	23.78	94.44	71.44	0.02	0.04	0.06	0.08	0.05
G-45	NIL-Thatcher-*Lr22a*-RL5404	1395.00	2475.00	227.50	637.50	1183.75	76.54	108.20	39.63	94.44	79.70	0.02	0.06	0.06	0.05	0.05
G-46	NIL-Thatcher-*Lr23*-LEE310	1687.50	2109.38	45.50	525.00	1091.84	92.59	92.21	7.93	77.78	67.63	0.05	0.05	0.02	0.04	0.04
G-47	NIL-Thatcher-*Lr24*-Agent	1395.00	2578.13	311.50	639.38	1231.00	76.54	112.70	54.27	94.72	84.56	0.02	0.04	0.09	0.08	0.06
G-48	Transec (Awned)-*Lr25*	1089.00	2118.75	45.50	643.13	974.09	59.75	92.62	7.93	95.28	63.90	0.04	0.01	0.02	0.07	0.04
G-49	NIL-Thatcher-*Lr26*-ST-1–25	1125.00	2175.00	154.00	637.50	1022.88	61.73	95.08	26.83	94.44	69.52	0.02	0.04	0.07	0.07	0.05
G-50	Gatcher	1215.00	1987.50	206.50	825.00	1058.50	66.67	86.89	35.98	122.22	77.94	0.02	0.05	0.08	0.08	0.06
G-51	NIL-Thatcher-*Lr29*-CS7 AG11	562.50	1368.75	84.00	570.00	646.31	30.86	59.84	14.63	84.44	47.44	0.02	0.07	0.04	0.01	0.03
G-52	NIL-Thatcher-*Lr30*-Tzio	1485.00	2381.25	206.50	453.75	1131.63	81.48	104.10	35.98	67.22	72.19	0.02	0.04	0.08	0.09	0.06
G-53	NIL-Thatcher-*Lr32*-Ae.ta	1192.50	2531.25	84.00	487.50	1073.81	65.43	110.66	14.63	72.22	65.74	0.02	0.03	0.04	0.01	0.03
G-54	NIL-Thatcher-*Lr33*-PI58548	1215.00	1550.63	381.50	487.50	908.66	66.67	67.79	66.46	72.22	68.28	0.02	0.03	0.10	0.03	0.05
G-55	NIL-Thatcher-*Lr34*-PI58548	1035.00	1215.00	136.50	562.50	737.25	56.79	53.11	23.78	83.33	54.25	0.02	0.05	0.06	0.04	0.04
G-56	NIL-Manitou-*Lr36*-T.SP2-9	1093.50	2343.75	276.50	600.00	1078.44	60.00	102.46	48.17	88.89	74.88	0.05	0.04	0.09	0.02	0.05
G-57	NIL-Thatcher-*Lr37*-VPM	922.50	1072.50	413.00	562.50	742.63	50.62	46.89	71.95	83.33	63.20	0.03	0.03	0.08	0.03	0.05
G-58	NIL-Thatcher-*Lrb*-Carina	1192.50	2681.25	395.50	675.00	1236.06	65.43	117.21	68.90	100.00	87.89	0.03	0.04	0.03	0.09	0.05
G-59	WL 711	1012.50	806.25	605.50	750.00	793.56	55.56	35.25	105.49	111.11	76.85	0.03	0.04	0.06	0.08	0.05
G-60	DW7276	1125.00	1087.50	276.50	682.50	792.88	61.73	47.54	48.17	101.11	64.64	0.02	0.04	0.09	0.09	0.06
G-61	Iumillo	1021.50	815.63	91.00	607.50	633.91	56.05	35.66	15.85	90.00	49.39	0.03	0.03	0.05	0.03	0.03
G-62	Local Red	1035.00	665.63	161.00	0.00	465.41	56.79	29.10	28.05	0.00	28.48	0.02	0.03	0.05	0.00	0.03
G-63	*Lr41*/6*TC	1395.00	2231.25	700.00	637.50	1240.94	76.54	97.54	121.95	94.44	97.62	0.02	0.03	0.03	0.02	0.03
G-64	TC*6/T.spelta 783	1530.00	240.00	553.00	566.25	722.31	83.95	10.49	96.34	83.89	68.67	0.03	0.05	0.06	0.03	0.04
G-65	TC/4 *ST-1	1080.00	215.63	136.50	525.00	489.28	59.26	9.43	23.78	77.78	42.56	0.03	0.04	0.06	0.03	0.04
G-66	Pavon + *Lr47*	1192.50	2081.25	542.50	562.50	1094.69	65.43	90.98	94.51	83.33	83.57	0.03	0.03	0.09	0.03	0.05
G-67	C 78.5	1282.50	1415.63	91.00	637.50	856.66	70.37	61.89	15.85	94.44	60.64	0.03	0.03	0.05	0.03	0.04
G-68	HP 348	387.00	1368.75	437.50	937.50	782.69	21.23	59.84	76.22	138.89	74.04	0.06	0.05	0.08	0.02	0.05
G-69	HPW 314	342.00	534.38	371.00	637.50	471.22	18.77	23.36	64.63	94.44	50.30	0.06	0.04	0.03	0.03	0.04
G-70	JAL95.4.3/3/Kachu # 1/Kiritati//Kachu	252.00	1096.88	206.50	487.50	510.72	13.83	47.95	35.98	72.22	42.49	0.04	0.02	0.06	0.03	0.04
G-71	JAL95.4.3/3/Kachu # 1/Kiritati//Kachu	342.00	1837.50	241.50	712.50	783.38	18.77	80.33	42.07	105.56	61.68	0.06	0.03	0.08	0.05	0.05
G-72	JAL95.4.3/3/Kachu # 1/Kiritati//Kachu	319.50	1258.13	136.50	0.00	428.53	17.53	55.00	23.78	0.00	24.08	0.05	0.02	0.06	0.00	0.03
G-73	JAL95.4.3/3/Kachu # 1/Kiritati//Kachu	1035.00	1903.13	339.50	675.00	988.16	56.79	83.20	59.15	100.00	74.78	0.02	0.02	0.03	0.03	0.02
G-74	JAL95.4.3/3/Kachu # 1/Kiritati//Kachu	1012.50	1275.00	451.50	787.50	881.63	55.56	55.74	78.66	116.67	76.65	0.03	0.02	0.08	0.01	0.04
G-75	JAL95.4.3/3/Kachu # 1/Kiritati//Kachu	1192.50	414.38	549.50	825.00	745.34	65.43	18.11	95.73	122.22	75.38	0.03	0.05	0.03	0.02	0.03
G-76	JAL95.4.3/3/Kachu # 1/Kiritati//Kachu	1507.50	592.50	430.50	900.00	857.63	82.72	25.90	75.00	133.33	79.24	0.05	0.07	0.04	0.03	0.05
G-77	JAL95.4.3/3/Kachu # 1/Kiritati//Kachu	1462.50	637.50	441.00	712.50	813.38	80.25	27.87	76.83	105.56	72.63	0.06	0.05	0.02	0.05	0.04
G-78	JAL95.4.3/3/Kachu # 1/Kiritati//Kachu	1507.50	487.50	472.50	787.50	813.75	82.72	21.31	82.32	116.67	75.75	0.05	0.04	0.01	0.06	0.04
G-79	JAL95.4.3/3/Kachu # 1/Kiritati//Kachu	1305.00	255.00	360.50	678.75	649.81	71.60	11.15	62.80	100.56	61.53	0.04	0.04	0.02	0.12	0.05
G-80	JAL95.4.3/3/Kachu # 1/Kiritati//Kachu	1215.00	1275.00	378.00	455.63	830.91	66.67	55.74	65.85	67.50	63.94	0.04	0.02	0.03	0.08	0.04
G-81	JAL95.4.3/3/Kachu # 1/Kiritati//Kachu	1363.50	1378.13	346.50	562.50	912.66	74.81	60.25	60.37	83.33	69.69	0.05	0.03	0.02	0.05	0.03
G-82	JAL95.4.3/3/Kachu # 1/Kiritati//Kachu	1003.50	232.50	497.00	528.75	565.44	55.06	10.16	86.59	78.33	57.54	0.05	0.05	0.02	0.10	0.05
G-83	JAL95.4.3/3/Kachu # 1/Kiritati//Kachu	1125.00	755.63	276.50	451.88	652.25	61.73	33.03	48.17	66.94	52.47	0.04	0.04	0.06	0.10	0.06
G-84	JAL95.4.3/3/Kachu # 1/Kiritati//Kachu	1440.00	898.13	241.50	637.50	804.28	79.01	39.26	42.07	94.44	63.70	0.03	0.02	0.06	0.03	0.04
G-85	JAL95.4.3/3/Kachu # 1/Kiritati//Kachu	1273.50	1083.75	231.00	637.50	806.44	69.88	47.38	40.24	94.44	62.99	0.05	0.03	0.05	0.03	0.04
G-86	JAL95.4.3/3/Kachu # 1/Kiritati//Kachu	1543.50	669.38	294.00	675.00	795.47	84.69	29.26	51.22	100.00	66.29	0.07	0.03	0.07	0.02	0.05
G-87	JAL95.4.3/3/Kachu # 1/Kiritati//Kachu	1363.50	652.50	45.50	637.50	674.75	74.81	28.52	7.93	94.44	51.43	0.05	0.02	0.02	0.01	0.02
G-88	JAL95.4.3/3/Kachu # 1/Kiritati//Kachu	1620.00	785.63	84.00	450.00	734.91	88.89	34.34	14.63	66.67	51.13	0.03	0.03	0.04	0.03	0.03
G-89	JAL95.4.3/3/Kachu # 1/Kiritati//Kachu	1462.50	442.50	385.00	450.00	685.00	80.25	19.34	67.07	66.67	58.33	0.03	0.06	0.07	0.03	0.05
G-90	JAL95.4.3/3/Kachu # 1/Kiritati//Kachu	1597.50	1063.13	0.00	450.00	777.66	87.65	46.48	0.00	66.67	50.20	0.05	0.03	0.00	0.03	0.03
G-91	JAL95.4.3/3/Kachu # 1/Kiritati//Kachu	1498.50	1035.00	119.00	525.00	794.38	82.22	45.25	20.73	77.78	56.49	0.06	0.04	0.04	0.02	0.04
G-92	IG 41514/5/Seri.1B//Kauz/Hevo/3/ Amad*2/4/Kiritati/6/Fret2 *2/4/SNI/ Trap#1/3/Kauz*2/…	1498.50	1546.88	45.50	675.00	941.47	82.22	67.62	7.93	100.00	64.44	0.06	0.03	0.02	0.03	0.03
G-93	CPI18/Gediz/3/Goo//ALB/CRA/4/ *AE. squarrosa* (494)/6/Kauz//Altar 84/…	1012.50	1950.00	45.50	675.00	920.75	55.56	85.25	7.93	100.00	62.18	0.03	0.04	0.02	0.02	0.03
G-94	PHSL 10	1080.00	669.38	416.50	637.50	700.84	59.26	29.26	72.56	94.44	63.88	0.02	0.04	0.04	0.03	0.03
G-95	PHSL 11	1417.50	483.75	315.00	750.00	741.56	77.78	21.15	54.88	111.11	66.23	0.05	0.03	0.07	0.03	0.04
G-96	AKW 4739	1485.00	425.63	84.00	412.50	601.78	81.48	18.61	14.63	61.11	43.96	0.02	0.03	0.04	0.02	0.03
G-97	GW 2010–272	1093.50	688.13	395.50	450.00	656.78	60.00	30.08	68.90	66.67	56.41	0.05	0.03	0.02	0.03	0.03
G-98	GW 2010–281	1620.00	609.38	427.00	382.50	759.72	88.89	26.64	74.39	56.67	61.65	0.03	0.03	0.02	0.05	0.03
G-99	GW 2010–281	1530.00	412.50	49.00	412.50	601.00	83.95	18.03	8.54	61.11	42.91	0.03	0.02	0.03	0.02	0.02
G-100	GW 2010–288	1552.50	598.13	262.50	937.50	837.66	85.19	26.15	45.73	138.89	73.99	0.02	0.03	0.06	0.02	0.03
G-101	VW 20145	1282.50	326.25	84.00	1080.00	693.19	70.37	14.26	14.63	160.00	64.82	0.03	0.05	0.04	0.02	0.04
G-102	VW 20167	1111.50	189.38	0.00	570.00	467.72	60.99	8.28	0.00	84.44	38.43	0.03	0.04	0.00	0.06	0.03
G-103	VW 20168	1201.50	515.63	154.00	607.50	619.66	65.93	22.54	26.83	90.00	51.32	0.03	0.05	0.04	0.07	0.05
G-104	EIGSN 2013–16	1102.50	853.13	521.50	785.63	815.69	60.49	37.30	90.85	116.39	76.26	0.03	0.05	0.04	0.03	0.04
G-105	EIGSN 2013–8	1111.50	547.50	630.00	375.00	666.00	60.99	23.93	109.76	55.56	62.56	0.03	0.04	0.02	0.03	0.03
G-106	EGSN 2013–13	855.00	768.75	521.50	637.50	695.69	46.91	33.61	90.85	94.44	66.45	0.02	0.04	0.04	0.03	0.03
G-107	NW 5054	1035.00	618.75	0.00	600.00	563.44	56.79	27.05	0.00	88.89	43.18	0.02	0.03	0.00	0.04	0.02
G-108	EIGSN 2013–36	1125.00	367.50	0.00	555.00	511.88	61.73	16.07	0.00	82.22	40.00	0.02	0.04	0.00	0.03	0.02
G-109	EIGSN 2013–55	1080.00	367.50	0.00	592.50	510.00	59.26	16.07	0.00	87.78	40.78	0.03	0.04	0.00	0.05	0.03
G-110	AKAW 4731	945.00	429.38	532.00	675.00	645.34	51.85	18.77	92.68	100.00	65.83	0.02	0.06	0.02	0.03	0.03
G-111	GW 281	787.50	480.00	476.00	637.50	595.25	43.21	20.98	82.93	94.44	60.39	0.02	0.05	0.02	0.02	0.03
G-112	VAS 320	697.50	603.75	0.00	600.00	475.31	38.27	26.39	0.00	88.89	38.39	0.02	0.04	0.00	0.03	0.02
G-113	ALY 1090	297.00	431.25	119.00	637.50	371.19	16.30	18.85	20.73	94.44	37.58	0.04	0.04	0.05	0.03	0.04
G-114	HE 1584	607.50	412.50	0.00	712.50	433.13	33.33	18.03	0.00	105.56	39.23	0.02	0.04	0.00	0.05	0.03
G-115	MP 1259	387.00	491.25	227.50	712.50	454.56	21.23	21.48	39.63	105.56	46.97	0.06	0.04	0.03	0.05	0.04
G-116	NP 3288	634.50	285.00	234.50	637.50	447.88	34.81	12.46	40.85	94.44	45.64	0.05	0.05	0.03	0.03	0.04
G-117	NIAW 34	387.00	618.75	189.00	637.50	458.06	21.23	27.05	32.93	94.44	43.91	0.06	0.03	0.07	0.03	0.05
G-118	NW 5013	342.00	249.38	136.50	0.00	181.97	18.77	10.90	23.78	0.00	13.36	0.04	0.05	0.06	0.00	0.04
G-119	PHS 1101	319.50	421.25	280.00	675.00	423.94	17.53	18.42	48.78	100.00	46.18	0.05	0.06	0.07	0.04	0.06
G-120	Axminister	0.00	90.00	388.50	412.50	222.75	0.00	3.93	67.68	61.11	33.18	0.00	0.04	0.03	0.02	0.02
G-121	Ulka	0.00	67.50	434.00	450.00	237.88	0.00	2.95	75.61	66.67	36.31	0.00	0.04	0.03	0.04	0.03
G-122	Asosan	0.00	93.75	0.00	382.50	119.06	0.00	4.10	0.00	56.67	15.19	0.00	0.05	0.00	0.05	0.03
G-123	Chul	0.00	33.75	0.00	412.50	111.56	0.00	1.48	0.00	61.11	15.65	0.00	0.04	0.00	0.02	0.01
G-124	Sonora	0.00	71.25	0.00	937.50	252.19	0.00	3.11	0.00	138.89	35.50	0.00	0.04	0.00	0.03	0.02
G-125	Syros	0.00	67.50	0.00	345.00	103.13	0.00	2.95	0.00	51.11	13.52	0.00	0.04	0.00	0.07	0.03
G-126	Khapli	0.00	99.38	336.00	0.00	108.84	0.00	4.34	58.54	0.00	15.72	0.00	0.03	0.04	0.00	0.02
G-127	Hope	868.50	350.63	507.50	570.00	574.16	47.65	15.33	88.41	84.44	58.96	0.03	0.06	0.10	0.07	0.06
G-128	Talent	0.00	465.00	0.00	375.00	210.00	0.00	20.33	0.00	55.56	18.97	0.00	0.05	0.00	0.03	0.02
G-129	Mercato//Parus/Pastor	652.50	830.63	84.00	175.00	435.53	35.80	36.31	14.63	25.93	28.17	0.09	0.03	0.04	0.08	0.06
G-130	Quaiu #1*2/Munal #1	382.50	922.50	682.50	406.00	598.38	20.99	40.33	118.90	60.15	60.09	0.09	0.02	0.03	0.06	0.05
G-131	Quaiu #1*2/Munal #1	693.00	1239.38	378.00	574.00	721.09	38.02	54.18	65.85	85.04	60.77	0.07	0.04	0.03	0.07	0.05
G-132	Urbina S2007*2/3/Guam92/ Kauz//Zhengyou 6	247.50	815.63	399.00	591.50	513.41	13.58	35.66	69.51	87.63	51.59	0.06	0.03	0.03	0.08	0.05
G-133	PRL/2*Pastor//Kachu	517.50	438.75	413.00	220.50	397.44	28.40	19.18	71.95	32.67	38.05	0.08	0.06	0.03	0.07	0.06
G-134	Sokoll/3/Pastor//HXL7573/2* BAU*2/4/NAVJ07	225.00	611.25	290.50	262.50	347.31	12.35	26.72	50.61	38.89	32.14	0.04	0.03	0.02	0.07	0.04
G-135	Babax/*Lr42*//Babax*2/3/Pavon7S3, + *Lr47*/4/Rolf07/Yanac//Tacupeto F2001/Brambling	652.50	673.13	339.50	287.00	488.03	35.80	29.43	59.15	42.52	41.72	0.06	0.03	0.03	0.09	0.05
G-136	Fret2/Kukuna//Fret2/3/Yanac/4/Fret2 /Kiritati*2/5/Whear//2*PRL/2*Pastor	328.50	922.50	308.00	262.50	455.38	18.02	40.33	53.66	38.89	37.72	0.08	0.05	0.03	0.07	0.06
G-137	ND643/2*Wbll1//Villa Juarez F2009	607.50	879.38	217.00	367.50	517.84	33.33	38.44	37.80	54.44	41.01	0.09	0.03	0.05	0.07	0.06
G-138	Mutus*2//TAM200/Turaco	652.50	575.63	374.50	381.50	496.03	35.80	25.16	65.24	56.52	45.68	0.07	0.03	0.01	0.08	0.05
G-139	Mutus*2//Haril #1	427.50	826.88	136.50	269.50	415.09	23.46	36.15	23.78	39.93	30.83	0.08	0.03	0.06	0.07	0.06
G-140	Mutus*2/Haril #1	427.50	1078.13	322.00	448.00	568.91	23.46	47.13	56.10	66.37	48.26	0.08	0.03	0.04	0.09	0.06
G-141	Quaiu/Becard//Becard	630.00	768.75	574.00	374.50	586.81	34.57	33.61	100.00	55.48	55.91	0.07	0.03	0.04	0.08	0.05
G-142	Check Lehmi	1822.50	2287.50	574.00	675.00	1339.75	100.00	100.00	100.00	100.00	100.00	0.06	0.06	0.06	0.02	0.05

* Malan = Malan (2016–17); Kukumseri = Kukumseri (2016); Palampur-17- = Palampur (2016–17); Palampur-18- = Palampur (2017–18)

### Genotypes with combined resistance to PM and YR

Among the 142 genotypes, three genotypes viz., Pollmer/CTY88.547, Syros and Talent, exhibited combined resistance with mean PM and YR disease severity < 10%. Fourteen genotypes i.e. G-65, G-69, G-72, G-75, G-82, G-85, G-87, G-89, G-110, G-113, G-116, G-119, G-133 and G134, developed mean PM and YR severity of < 20%, were moderately resistant (Table 2 and 3).

## Discussion

Growing disease-resistant wheat cultivars represents a preferred strategy for managing powdery mildew (PM) and yellow rust (YR), primarily due to its cost-effectiveness, environmental sustainability, and benefits for agricultural practitioners. Consequently, the development of resistance to YR and PM has become a principal objective for both the breeders and plant pathologists [44]. Nevertheless, the majority of commercially cultivated wheat varieties in India’s epidemiologically significant North Hill Zone and North Western Plain Zone (NWPZ) exhibit susceptibility to both the diseases, as indicated in the Annual Report of the Indian Institute of Wheat and Barley Research (IIWBR) for the period 2023–24 and earlier period. The rapid emergence of new pathotypes, combined with the complex nature of host pathogen interactions and various environmental factors, has considerably complicated the identification of diverse and stable resistance donors [45] for ongoing evergreen breeding programs on a global scale. While field evaluations serve as practical methodologies to identify resistant donors for PM and YR, these evaluations are vulnerable to unpredictable environmental conditions and fluctuations in pathogen populations [2, 7, 46, 47]. In the light of these challenges, the present study was initiated to identify genotypes exhibiting stable resistance among 142 diverse wheat genotypes for PM and YR across four and three distinct hotspot locations, respectively, which represent various agro-climatic zones of North Western Himalayas as well as India. The study aims to delineate genotypes that demonstrated significant resistance levels with minimal variability across different locations.

Several studies have suggested that simple ANOVA is not enough for multi-location experimental studies as it does not account for the contribution of the genotypes to the environmental interaction, nor identify the genotypes with stable resistance across diverse environments [1, 17, 41, 43]. Among various statistical methods, AMMI and GGE biplot analyses have been reported as desirable tools for identifying disease resistant, stable genotypes under specific or across environments, as well as for determining hotspot locations for disease outbreaks [1]. Both AMMI and GGE biplot analyses have been used widely to evaluate crop genotypes for resistance to various diseases [1, 7, 47–50].

The analysis using the AMMI model indicated that for PM severity, the sum of squares attributable to the GEI surpassed that of both the E and G. This finding suggested significant variability in genotypic responses across different environments. The results are consistent with previous studies on various crop diseases [49, 51, 52]. Regarding YR, the sum of squares for G was more significant than those for GEI and E, thereby underscoring considerable diversity among genotypes. Furthermore, it was determined that the first two principal components (PCs) accounted for 85.53% of the GEI variance for PM and 100% for YR. This observation indicated that a significant portion of the variation was captured by these two components, which aligns with prior research on soybean yield stability, Fusarium head blight and stem rust in wheat [1, 52, 53]. It is recommended that the most accurate AMMI model be derived from the first two interaction principal component axes (IPCA), as suggested in earlier literature [43, 54].

The AMMI model has proven effective, as it accounted for a significant portion of the GEI sum of squares and successfully separated the main and interaction effects. Similarly, the GGE biplot model is valuable for identifying stable cultivars across environments and determining the best-performing cultivars for specific mega-environments [15]. The findings demonstrated that both the AMMI and GGE biplot models yielded comparable results in terms of specific adaptability to environmental conditions. The AMMI 1 biplot identified E2 and E4 as highly conducive environments for PM development, and En1 and En3 as favourable for YR. The AMMI 2 biplot highlighted variability among environments—specifically E1, E2, and E3 for PM, and En1, En2, and En3 for YR—based on their distances from the origin, indicating differences in disease severity across locations.

The GGE biplot offers a graphical representation of the “which-won-where” configuration for easy identification of environments with consistent “winning” genotypes [1]. Mega environments (MEs) are groups of locations that show consistent genotype performance when tested with the same set of genotypes [40, 41, 45]. Identifying these MEs is essential for understanding genotype-by-environment interaction (GEI) and improving selection for targeted adaptations. Currently, two MEs were identified for PM and YR, grouping four and three test locations, respectively, each with distinct winning genotypes—indicating the presence of crossover GEI. Information on MEs enables breeders to identify the discriminating and representative environments, making them valuable test sites for detecting broadly adapted genotypes or for breeding genotypes tailored to specific environmental conditions [16]. The GGE biplot method is commonly employed to visually identify that which genotypes perform best within specific MEs [50, 55]. Those genotypes close to the environment vectors and inside sector of a particular environment or group of environments were well adopted in that environment (showed high disease severity of PM and YR). In the current study, genotypes G-41, G-45, G-46, and G-53 demonstrated strong adaptation to the E2 sector for PM, while genotypes G-1, G-2, G-10, and G-11 were well adapted to environments En1 and En3.

The cosine of the angles between the test environment vectors (i.e., the angle between the test environment and a target environment) represented the genetic association between them [47, 56]. Small (< 90°) and large angles (> 90°) between environment vectors indicated strong positive and negative correlation, respectively [40, 43]. A strong positive relationship was observed between E3 and E4, for PM and between En1 and En3 for YR, where the genotypes displayed similar disease reactions.

Test environment with long vector lengths were considered as discriminating, whereas, test environments with small angles with “AEC abscissa” indicated representativeness [56, 57]. In the present study “discriminativeness vs representativeness” view of the biplot of test environments showed that E2 and En1 with the longest vector length demonstrated high discriminating power and had greater angle with the AEC abscissa, indicating its low representativeness due to the highest PM and YR disease pressure, respectively. Therefore, these environments are suitable for identifying the most disease-resistant genotypes.

Genotype stability, evaluated using the AMMI Stability Value (ASV), revealed significant variation among genotypes across different environments. Genotypes with ASV values exceeding 3 for PM and 7 for YR were classified as unstable in their disease response across environments. Conversely, genotypes with ASV values close to 0 for both PM and YR were considered to exhibit stable disease severity—whether high or low—across all test locations. In the current study, genotypes G-13, G-62, G-7, G-81, G-112 and G-136 had ASV values near 0, indicating stable PM disease severity across the test sites. Similarly, genotypes G-51, G-125, G-23, G-106, G-83, G-92, G-84, G-90, G-131, G-82, G-68 and G-128 displayed consistent YR disease severity across all locations. These findings align with those of Mengesha [1] and Panda et al. [58], who reported that genotypes with higher ASV values tend to exhibit variable disease reactions depending on the location. The AMMI 1 and GGE (“which-won-where”) biplot showed that 10 and 37 wheat genotypes, located farthest to the left side of the biplot origin, exhibited the lowest PM and YR disease severity, respectively. These findings corroborated with the earlier workers [2, 41, 50, 51] who reported that the vertex or farthest genotypes from biplot origin were either resistant in specific or across the environments and could serve as valuable donors for PM and YR resistance in wheat-breeding programs. Conversely, genotypes that showed similar levels of disease across locations were positioned closely on AMMI 2 and GGE biplot origin. These findings were in alignment with the earlier reports of [51, 58]

A specific genotype that demonstrates lower severity of PM and YR at one location may exhibit differing levels of disease severity at other locations. This variation may be ascribed to the quantity and potential of inoculum load available in a particular environment and the prevailing environmental conditions—particularly temperature and humidity—favorable to pathogen infection. The interaction among these conducive ecological factors, the presence of susceptible host plants, and virulent pathogens during the epidemic period markedly influenced disease dynamics [59–61]. The present study observed the highest mean severity of PM at Kukumseri (E2) during the summer of 2016. This observation can be attributed to the pathogen's capability to persist as viable cleistothecia/chasmothecia (teleomorph) in the dry temperate zone of Himachal Pradesh, India. These structures undergo sexual recombination and meiosis, maturing into asci and producing ascospores significantly earlier in the season. This early maturation certainly results in pathogenically diverse and more virulent isolates [62].

The observed inconsistency in YR severity across various locations may result from the earlier evolution of pathotypes of the pathogen in the Northwest Himalayan Zone. This can also be linked to the survival of the *Pst* pathogen on volunteer plants or crops cultivated during the summer off-season, particularly in dry temperate regions such as Lahaul and Spiti. Furthermore, this area has been recognized as a hotspot and a primary centre for YR to other areas of the northwestern Himalayas, including Himachal Pradesh [63].

Ten and 37 genotypes showed resistance to PM and YR, respectively. Moreover, 20 genotypes were moderately resistant to PM at seedling stage. As observed in the present study, several workers have reported resistance to PM at the seedling and adult plant stage in India [2, 7, 23, 64–69] and abroad [64, 70–73]. Likewise, adult plant resistance to YR in Indian wheat genotypes has been identified and characterized in previous studies [2, 69, 70, 74, 75]. Furthermore, Vikarm et al. [76] identified resistance to YR and stem rust in Mexican wheat landraces.

Adult plant resistance (APR), also known as horizontal resistance, to PM reduces infection and pathogen reproduction by influencing key stages in the pathogen’s infection cycle including, initiation of symptoms, germination of spore, penetration, colonization, and sporulation. This form of stable resistance persists in cultivars regardless of the presence of race-specific genes and remains effective over time, as was observed in cv. Knox and its derivatives, such as Massey [73]. In the present study, 32 genotypes showed slow mildewing resistance to PM at adult plant stage. According to, GRIS (Accessions List (wheatpedigree.net)), 9 genotypes have been reported to carry known *Pm* resistant genes i.e. Axminister (*Pm1a*), Ulka (*Pm2*), Asosan (*Pm3a*), Chul (*Pm3b*), Sonora (*Pm3c*), Khapli (*Pm4a*), Talent (*Pm5* + *?*), PRL/2*Pastor//Kachu (*Pm4b*), and Sokoll/3/Pastor//HXL7573/2*BAU*2/4/NAVJ07 (*Pm4b*) (Table 3). Several studies have reported that *Pm* genes viz. *Pm3a*, *Pm3b*, *Pm3c*, *Pm7*, *Pm8* and *Pm17* exhibited rate reducing resistance, characterized by longer incubation and/or latent periods, smaller colony size and lower sporulation compared to susceptible cultivars like Agra Local [64, 70]. In India, the virulence against *Pm2* has remained stable over the past two decades [6, 7]. In contrast, pathogen populations showed an increase in virulence on gene *Pm4b* during the same period [6, 77, 78]. While many of these resistance genes were knocked down by new virulences, but such genes continue to provide residual resistance, as inferred by lower disease severity, AUDPC, rAUDPC and infection rates. This phenomenon has been observed in the present studies in genotypes G-21, G-25, G-62, G-65, G-70, G-72, G-96, G-99, G-102, G-107, G-108, G-109, G-112, G-113, G-114, G-115, G-116, G-117, G-118, G-119, G-120, G-121, G-122, G-123, G-124, G-125, G-126, G-128, G-133 and G-134. These genotypes showed susceptible reaction at seedling stage but were resistant at adult plant stage. Such type of observations was corroborated by earlier work also [2, 7]. Quantitative trait loci (QTLs) associated with APR were reported to be located near to these defeated major genes i.e. *Pm4*, *Pm5* and *Pm6* [78] and possibly might have contributed to residual resistance effects.

Various *Yr* and *Lr* resistant genes were postulated in six test genotypes used in the present study i.e. Seri M 82 (*Yr 2, Yr 9, Yr 29, Yr30* +), Local Red (*Lr14a, Lr18*), NW 5054 (*Lr23* +, *Yr2* +), NIAW 34 (*Lr13*, *Lr26*, *Lr34*, *Yr18*), Prl/2*Pastor//Kachu (*Yr29, Yr30, Yr31*) and Sokoll/3/Pastor//Hxl7573/2*Bau*2/4/Navj07 (*Lr26, Yr9, Yr29, Yr30, Yr31*) (Table 3) (GRIS (Accessions List (wheatpedigree.net)). The gene *Yr9* along with genes *Yr2*, *Yr18*, *Yr27*, *Yr30* and other unidentified APR yellow rust resistant genes, has contributed to YR resistance in India [79, 80]. Among the YR resistance genes, *Yr18* and *Yr30* confer non-specific APR, while *Yr9* and *Yr27* offered race-specific resistance to YR [80, 81]. For leaf rust (LR) resistance, *Lr13* and *Lr34* confered APR, while *Lr23*, *Lr24*, and *Lr26* provide race-specific resistance [80]. *Lr34* (= *Yr18/Sr57/Pm38*) is associated with partial APR to *Puccinia triticina* and genotypes carrying this gene showed improved resistance to YR [82], stem rust [83], and PM [84–87].

The slow mildewing resistance observed in the test genotypes may be attributed to the presence of YR resistance genes *Yr9*, *Yr18*, *Yr29* and *Yr30* and LR resistance genes *Lr13*, *Lr14a, Lr18, Lr23* + *, Lr26* and *Lr34* which could be linked to various known or unknown PM resistance genes. Various studies have reported that genes such as *Yr18/Lr34/Sr57/Pm38* [88, 89], *Yr29/Lr46/Sr58/Pm39* [90], *Yr30/Lr27/Pm48/Sr2* [91] and *Sr31/Yr9/Lr26/Pm9* [92] and *Lr34/Yr18/Sr57/Pm38/Ltn1* [93] have conferred resistance against several biotrophic pathogens.

Three genotypes, Pollmer/CTY88.547, Syros and Talent showed combined resistance to PM and YR and 14 genotypes with mean PM and YR disease severity of < 20% were moderately resistant. The present studies of genotypes with combined resistance to YR and PM were in agreement with earlier studies [2, 68, 94, 95].

E2 and En1 have been recognized as effective environments for the assessment and selection of superior wheat genotypes concerning their resistance against PM and YR, respectively. The present study seeks to elucidate the influence of environmental factors and genotype-environment interactions (GEI) on the responsiveness of wheat genotypes to PM and YR. The observed inconsistencies in genotype performance across different environments underscore the significant role of environmental variables in influencing the variability of PM and YR diseases. The differences noted among the tested genotypes across various environments indicate GEI's existence.

## Conclusion

The study revealed substantial variability in resistance to PM and YR among wheat genotypes evaluated across multiple environments. AMMI and GGE biplot analysis highlighted significant genotype-by-environment interaction (GEI), reflecting inconsistent resistance levels across environments. Among the 142 genotypes tested, ten genotypes exhibited high level of resistance to PM, thirty seven were resistant to YR, and 30 displayed slow mildewing resistance at the adult plant stage. The genotypes Pollmer/CTY88.547, Syros, and Talent (*Pm5* + *?*) emerged as the most promising, combining resistance to both PM and YR making them valuable for breeding and direct cultivation, pending agronomic validation. E2 and En1 were identified as optimal environments for selecting superior resistant genotypes for PM and YR, respectively. The identification of “Mega-environment” facilitated a reorganization of agro-ecological regions and breeding locations, enabling more efficient resource utilization. Consequently, suitable genotypes for specific environments can be developed and cultivated using optimized cultural practices to mitigate PM and YR impacts and reduce yield losses.

## Supplementary Information

Supplementary Material 1.

## Data Availability

No datasets were generated or analysed during the current study.
