# Supplementary material for: Genotype by environment interaction analysis for resistance against powdery mildew and yellow rust in some promising exotic wheats
Source: BMC Plant Biol. 2025 Jul 1;25:786. doi: 10.1186/s12870-025-06788-0 (PMC12211907; doi:10.1186/s12870-025-06788-0)
Supplement: Supplementary file 1 — Supplementary Material 1. [file 12870_2025_6788_MOESM1_ESM.docx]

**Table-S1**

Interaction principal component (IPCA1 and IPCA2) value of 142 wheat genotypes tested against PM and YR at CSKHPKV Palampur rabi (2016-17, 2017-2018), HAREC Kukumseri (summer 2016), Keylong (summer 2017) and RWRC Malan (2016-17).

|  |  | **Powdery mildew (PM)** | | | **Yellow rust (YR)** | | |
| --- | --- | --- | --- | --- | --- | --- | --- |
| **S. No.** | **Genotypes** | **IPCA1** | **IPCA2** | **ASV** | **IPCA1** | **IPCA2** | **ASV** |
| G-1 | Morocco | -0.13 | -0.25 | 1.16 | -0.90 | -0.20 | 10.32 |
| G-2 | Avocet-*Yra* | 0.02 | -0.79 | 2.51 | -0.34 | 0.26 | **3.98** |
| G-3 | Avocet+*Yra* | 0.02 | -0.79 | 2.51 | 0.40 | 0.48 | **4.97** |
| G-4 | *Yr1*/6*AOC | -0.31 | -0.77 | 3.21 | 0.72 | 0.87 | 8.94 |
| G-5 | Siete Cerros T66 | 0.10 | 0.01 | 0.64 | 0.87 | 0.04 | 2.91 |
| G-6 | Tatara (*Yr 3,Yr 29+*) | -0.19 | 0.35 | 1.68 | -0.08 | -0.10 | 0.97 |
| G-7 | *Yr5*/6*AOC | -0.01 | -0.08 | 0.25 | 0.09 | -0.12 | 1.11 |
| G-8 | *Yr6*/6*AOC | -0.17 | -0.26 | 1.43 | -0.67 | 0.31 | **7.73** |
| G-9 | *Yr7*/6*AOC | -0.14 | -0.20 | 1.14 | -0.50 | 0.07 | **5.67** |
| G-10 | *Yr8*/6*AOC | -0.40 | -0.28 | 2.83 | -0.71 | 0.37 | 8.26 |
| G-11 | *Yr9*/6*AOC | -0.02 | 0.17 | 0.54 | -0.79 | 0.05 | 8.96 |
| G-12 | *Yr10*/6*AOC | -0.23 | -0.14 | 1.59 | 0.15 | 0.00 | 1.71 |
| G-13 | *Yr15*/6*AOC | 0.01 | 0.03 | 0.12 | 0.17 | -0.02 | 1.91 |
| G-14 | *Yr17*/6*AOC | 0.16 | 0.11 | 1.11 | -0.16 | 0.02 | 2.87 |
| G-15 | *Yr18*/3*AOC | -0.15 | -0.37 | 1.54 | -0.20 | 0.38 | 2.70 |
| G-16 | *Yr24*/3*AOC | 0.60 | -0.30 | 4.18 | 0.00 | 0.22 | **0.87** |
| G-17 | *Yr26*/3*AOC | **0.69** | 0.22 | 4.70 | 0.08 | 0.10 | 1.00 |
| G-18 | *Yr27*/6*AOC | -0.36 | 0.17 | 2.51 | 0.35 | 0.76 | 5.05 |
| G-19 | *YrSP*/6*AOC | 0.17 | -0.60 | 2.19 | 0.64 | 0.15 | 3.35 |
| G-20 | Pavon F 76 (*Yr 6, Yr 7, Yr 29, Yr 30+*) | 0.02 | 0.31 | 0.98 | 0.08 | -0.31 | 1.54 |
| G-21 | Seri M 82 (*Yr 2, Yr 9, Yr 29, Yr 30+*) | 0.05 | -0.43 | 1.40 | 0.30 | -0.20 | 3.48 |
| G-22 | Opata M 85 (*Yr 27, Yr 18, Yr 30 +*) | **0.65** | 0.29 | 4.47 | -0.16 | 0.02 | **2.87** |
| G-23 | Super Kauz (*Yr 9, Yr 27, Yr 18, Yr30+*) | 0.36 | 0.20 | 2.53 | 0.04 | -0.06 | 0.56 |
| G-24 | *Yr*cv/6*AOC | 0.36 | 0.20 | 2.53 | 0.30 | 0.52 | 3.99 |
| G-25 | AOC-*Yr**3/3/Altar 84/Ae.sq//Opata | -0.23 | 0.22 | 1.72 | 0.12 | 0.04 | 2.42 |
| G-26 | AOC-YR*3//Lalbmon O1 *4/PVN | **0.70** | 0.07 | 4.71 | 0.24 | 0.29 | 3.99 |
| G-27 | AOC-*Yr**3/Pastor | 0.26 | 0.23 | 2.93 | 0.27 | 0.40 | 3.44 |
| G-28 | Pollmer/CTY88.547 | -0.38 | -0.07 | 2.60 | 0.17 | -0.02 | 1.91 |
| G-29 | Thatcher | 0.26 | 0.46 | 2.29 | 0.54 | 0.09 | 6.13 |
| G-30 | NIL-Thatcher-*Lr1*-CTR | 0.30 | 0.41 | 2.38 | 0.67 | -0.09 | 7.60 |
| G-31 | NIL-Thatcher-*Lr2a*-WST | 0.44 | 0.21 | 3.02 | 0.28 | 0.45 | 3.65 |
| G-32 | NIL-Thatcher-*Lr2b*-Carina | 0.29 | 0.13 | 2.02 | 0.14 | 0.11 | 2.69 |
| G-33 | NIL-Thatcher-*Lr2c*-Loros | 0.57 | 0.44 | 4.08 | 0.08 | -0.31 | 1.54 |
| G-34 | NIL-Thatcher-*Lr3*-Democrat | 0.28 | 0.28 | 2.09 | 0.32 | -0.24 | 3.81 |
| G-35 | IL-Thatcher-*Lr3*KA-AIV | 0.12 | 0.05 | 0.86 | -0.41 | -0.05 | 4.68 |
| G-36 | NIL-Thatcher-*Lr3*BG-Bage | 0.49 | 0.48 | 3.63 | -0.24 | -0.29 | 3.96 |
| G-37 | NIL-Thatcher-*Lr9*-Tranfer | 0.36 | 0.41 | 2.79 | -0.08 | -0.10 | 0.97 |
| G-38 | Hussar | 0.44 | 0.40 | 3.21 | -0.16 | -0.20 | 2.96 |
| G-39 | NIL-Thatcher-*Lr12*-EX | **0.69** | 0.01 | 4.69 | -0.37 | 0.10 | 4.28 |
| G-40 | Manitou | 0.31 | 0.23 | 2.24 | -0.34 | 0.26 | 3.98 |
| G-41 | RL 6006 | **0.68** | 0.46 | 4.81 | -0.29 | -0.02 | 3.28 |
| G-42 | NIL-Thatcher-*Lr16*-EX | 0.56 | 0.22 | 3.86 | -0.25 | 0.14 | 2.91 |
| G-43 | NIL-Thatcher-*Lr19*-TR | 0.51 | 0.08 | 3.47 | -0.50 | 0.07 | 5.67 |
| G-44 | NIL-Thatcher-*Lr21*-RL5406 | 0.56 | -0.12 | 3.80 | -0.37 | 0.10 | 4.28 |
| G-45 | NIL-Thatcher-*Lr22a*-RL5404 | **1.00** | -0.20 | 6.78 | -0.32 | -0.17 | 3.76 |
| G-46 | NIL-Thatcher-*Lr23*-LEE310 | **0.78** | 0.20 | 5.32 | -0.50 | 0.07 | 5.67 |
| G-47 | NIL-Thatcher-*Lr24*-Agent | 0.57 | -0.53 | 4.17 | -0.37 | 0.10 | 4.28 |
| G-48 | Transec (Awned)-*Lr25* | 0.28 | 0.47 | 2.39 | -0.08 | 0.63 | 2.70 |
| G-49 | NIL-Thatcher-*Lr26*-ST-1-25 | 0.60 | -0.19 | 4.10 | 0.24 | 0.29 | 2.99 |
| G-50 | Gatcher | 0.45 | -0.23 | 3.09 | -0.12 | -0.26 | 2.66 |
| G-51 | NIL-Thatcher-*Lr29*-CS7AG11 | 0.47 | -0.15 | 3.23 | -0.02 | -0.08 | 0.37 |
| G-52 | NIL-Thatcher-*Lr30*-Tzio | **0.66** | -0.35 | 4.62 | -0.07 | -0.31 | 1.51 |
| G-53 | NIL-Thatcher-*Lr32*-Ae.ta | **0.71** | 0.13 | 4.82 | -0.46 | 0.22 | 5.32 |
| G-54 | NIL-Thatcher-*Lr33*-PI58548 | 0.05 | -0.69 | 2.21 | 0.01 | -0.43 | 2.76 |
| G-55 | NIL-Thatcher-*Lr34*-PI58548 | 0.22 | -0.05 | 1.49 | 0.08 | 0.09 | 0.97 |
| G-56 | NIL-Manitou-*Lr36*-T.SP2-9 | **0.62** | -0.56 | 4.54 | -0.25 | 0.14 | 2.91 |
| G-57 | NIL-Thatcher-*Lr37*-VPM | -0.10 | -0.31 | 1.22 | -0.08 | -0.10 | 0.97 |
| G-58 | NIL-Thatcher-*Lrb*-Carina | **0.69** | -0.28 | 4.74 | -0.03 | -0.37 | 2.55 |
| G-59 | WL 711 | -0.58 | -0.42 | 4.13 | -0.12 | -0.04 | 1.39 |
| G-60 | DW7276 | -0.18 | -0.32 | 1.60 | 0.17 | -0.02 | 1.91 |
| G-61 | Iumillo | -0.13 | 0.12 | 0.95 | 0.08 | 0.10 | 1.00 |
| G-62 | Local Red | -0.02 | -0.03 | 0.14 | -0.58 | 0.19 | 6.69 |
| G-63 | *Lr41*/6*TC | 0.39 | -0.50 | 3.09 | -0.32 | -0.17 | 3.76 |
| G-64 | TC*6/T.spelta 783 | -0.67 | -0.22 | 4.58 | -0.23 | 0.00 | 2.57 |
| G-65 | TC/4 *ST-1 | -0.47 | 0.25 | 3.28 | -0.01 | -0.30 | 1.20 |
| G-66 | Pavon + *Lr47* | 0.38 | -0.51 | 3.01 | -0.31 | 0.12 | 3.59 |
| G-67 | C 78.5 | 0.24 | 0.03 | 0.63 | -0.25 | 0.14 | 2.91 |
| G-68 | HP 348 | 0.34 | -0.38 | 2.61 | 0.04 | 0.16 | 0.76 |
| G-69 | HPW 314 | -0.35 | -0.10 | 2.37 | 0.15 | 0.00 | 1.71 |
| G-70 | JAL95.4.3/3/Kachu # 1/Kiritati//Kachu | 0.01 | -0.27 | 0.87 | -0.29 | -0.02 | 3.28 |
| G-71 | -do- | 0.33 | -0.34 | 2.48 | -0.29 | -0.02 | 3.28 |
| G-72 | -do- | 0.26 | -0.32 | 2.02 | -0.16 | 0.02 | 2.87 |
| G-73 | -do- | 0.23 | -0.01 | 1.57 | -0.21 | 0.30 | 2.72 |
| G-74 | -do- | -0.03 | -0.18 | 0.60 | -0.16 | 0.02 | 2.87 |
| G-75 | -do- | -0.42 | -0.13 | 2.83 | 0.08 | 0.10 | 1.00 |
| G-76 | -do- | -0.38 | 0.10 | 2.57 | 0.01 | -0.22 | 0.88 |
| G-77 | -do- | -0.30 | 0.33 | 2.30 | 0.01 | -0.22 | 0.88 |
| G-78 | -do- | -0.41 | 0.15 | 2.79 | 0.05 | -0.48 | 2.03 |
| G-79 | -do- | -0.54 | 0.46 | 3.92 | -0.32 | -0.17 | 3.76 |
| G-80 | -do- | -0.07 | 0.28 | 1.01 | -0.07 | -0.53 | 2.27 |
| G-81 | -do- | 0.03 | 0.07 | 0.27 | 0.07 | -0.20 | 1.31 |
| G-82 | -do- | -0.48 | -0.01 | 2.25 | 0.04 | 0.16 | 0.76 |
| G-83 | -do- | -0.32 | 0.48 | 2.65 | -0.02 | 0.14 | 0.61 |
| G-84 | -do- | -0.19 | 0.24 | 1.50 | 0.06 | 0.02 | 0.72 |
| G-85 | -do- | 0.13 | 0.01 | 0.88 | 0.01 | -0.22 | 0.88 |
| G-86 | -do- | -0.33 | 0.31 | 2.42 | 0.17 | -0.02 | 1.91 |
| G-87 | -do- | -0.17 | 0.21 | 1.31 | 0.07 | -0.30 | 1.44 |
| G-88 | -do- | -0.13 | 0.27 | 1.24 | 0.72 | 0.14 | 8.24 |
| G-89 | -do- | -0.31 | -0.06 | 2.06 | 0.08 | 0.08 | 0.94 |
| G-90 | -do- | 0.03 | 0.39 | 1.26 | 0.06 | 0.02 | 0.72 |
| G-91 | -do- | -0.03 | 0.32 | 1.02 | -0.03 | -0.37 | 1.55 |
| G-92 | IG 41514/5/Seri.1B//Kauz/ Hevo/3/Amad*2 /4/ Kiritati /6/Fret2 *2/4/ SNI/ Trap#1 /3/Kauz*2/… | 0.26 | 0.32 | 2.05 | 0.06 | 0.02 | 0.72 |
| G-93 | CPI18/Gediz/3/Goo//ALB/CRA/4/ *AE. squarrosa* (494)/6/ Kauz//Altar 84/… | 0.53 | 0.10 | 3.96 | -0.34 | 0.26 | 3.98 |
| G-94 | PHSL 10 | -0.26 | -0.16 | 1.82 | 0.75 | 0.00 | 8.51 |
| G-95 | PHSL 11 | -0.34 | 0.08 | 2.28 | 0.09 | -0.34 | 2.73 |
| G-96 | AKW 4739 | -0.28 | 0.13 | 1.90 | 0.17 | -0.02 | 2.91 |
| G-97 | GW 2010-272 | -0.16 | 0.00 | 1.05 | 0.25 | -0.14 | 2.95 |
| G-98 | GW 2010-281 | -0.25 | 0.08 | 1.23 | 0.18 | -0.46 | 2.76 |
| G-99 | GW 2010-281 | -0.34 | 0.38 | 2.61 | 0.08 | 0.10 | 3.00 |
| G-100 | GW 2010-288 | -0.29 | 0.22 | 2.05 | 0.08 | 0.10 | 3.00 |
| G-101 | VW 20145 | -0.27 | 0.11 | 1.25 | 0.25 | -0.14 | 2.95 |
| G-102 | VW 20167 | -0.36 | 0.27 | 2.55 | 0.76 | -0.42 | 8.77 |
| G-103 | VW 20168 | -0.20 | 0.13 | 1.40 | 0.92 | -0.24 | 10.52 |
| G-104 | EIGSN 2013-16 | -0.09 | -0.21 | 0.91 | 1.00 | -0.35 | 11.47 |
| G-105 | EIGSN 2013-8 | -0.36 | -0.16 | 2.48 | 0.61 | 0.40 | 7.14 |
| G-106 | EGSN 2013-13 | -0.21 | -0.18 | 1.56 | 0.04 | -0.06 | 3.56 |
| G-107 | NW 5054 | -0.07 | 0.18 | 0.71 | -0.29 | -0.02 | 3.28 |
| G-108 | EIGSN 2013-36 | -0.33 | 0.31 | 2.45 | -0.40 | -0.49 | 4.94 |
| G-109 | EIGSN 2013-55 | -0.41 | 0.52 | 3.19 | -0.15 | -0.41 | 2.40 |
| G-110 | AKAW 4731 | -0.31 | -0.08 | 2.10 | -0.07 | -0.53 | 2.27 |
| G-111 | GW 281 | -0.21 | -0.29 | 1.70 | -0.15 | -0.41 | 2.40 |
| G-112 | VAS 320 | -0.05 | 0.02 | 0.37 | 0.09 | -0.34 | 2.73 |
| G-113 | ALY 1090 | -0.18 | -0.17 | 1.32 | 0.30 | -0.41 | 3.79 |
| G-114 | HE 1584 | -0.23 | 0.12 | 1.61 | -0.41 | -0.05 | 4.68 |
| G-115 | MP 1259 | -0.28 | 0.06 | 1.89 | -0.32 | -0.17 | 3.76 |
| G-116 | NP 3288 | -0.32 | -0.12 | 2.22 | -0.16 | 0.02 | 2.87 |
| G-117 | NIAW 34 | -0.24 | -0.16 | 1.72 | -0.42 | 0.38 | 5.06 |
| G-118 | NW 5013 | -0.21 | -0.31 | 1.71 | -0.37 | 0.10 | 4.28 |
| G-119 | PHS 1101 | -0.30 | -0.21 | 2.15 | -0.08 | -0.10 | 0.97 |
| G-120 | Axminister | -0.35 | -0.33 | 2.57 | 0.07 | 0.53 | 2.27 |
| G-121 | Ulka | -0.41 | -0.43 | 2.10 | -0.55 | 0.34 | **6.38** |
| G-122 | Asosan | -0.23 | -0.14 | 1.59 | -0.20 | -0.14 | 2.36 |
| G-123 | Chul | -0.30 | -0.13 | 2.06 | 0.00 | 0.22 | 2.87 |
| G-124 | Sonora | -0.36 | -0.05 | 2.40 | 0.24 | 0.29 | 2.99 |
| G-125 | Syros | -0.32 | -0.08 | 2.14 | 0.04 | -0.06 | 0.56 |
| G-126 | Khapli | -0.27 | -0.39 | 2.21 | -0.29 | 0.20 | 3.44 |
| G-127 | Hope | -0.58 | -0.72 | 4.51 | 0.53 | 0.52 | 6.33 |
| G-128 | Talent | -0.09 | -0.13 | 0.73 | -0.01 | 0.20 | 0.82 |
| G-129 | Mercato//Parus/Pastor | -0.11 | 0.44 | 1.58 | 0.14 | 0.01 | 1.61 |
| G-130 | Quaiu #1*2/Munal #1 | -0.45 | 0.14 | 3.03 | 0.09 | -0.12 | 1.11 |
| G-131 | Quaiu #1*2/Munal #1 | -0.14 | 0.36 | 1.45 | 0.06 | 0.02 | 0.72 |
| G-132 | Urbina S2007*2/3/ Guam92/ Kauz//Zhengyou 6 | -0.32 | 0.04 | 2.17 | 0.13 | -0.08 | 1.54 |
| G-133 | PRL/2*Pastor//Kachu | -0.36 | 0.11 | 2.42 | 0.09 | -0.12 | 1.11 |
| G-134 | Sokoll/3/Pastor//HXL7573/2*BAU*2/4/NAVJ07 | -0.24 | -0.05 | 1.81 | 0.12 | -0.07 | 1.43 |
| G-135 | Babax/*Lr42*//Babax*2/3/Pavon7S3,+*Lr47*/4/Rolf07/ Yanac//Tacupeto F2001/ Brambling | -0.30 | 0.33 | 2.27 | 0.11 | -0.16 | 1.44 |
| G-136 | Fret2/Kukuna//Fret2/3/Yanac/4/Fret2/Kiritati*2/5/Whear//2*PRL/2*Pastor | -0.02 | -0.11 | 0.39 | 0.11 | -0.04 | 1.22 |
| G-137 | ND643/2*Wbll1//Villa Juarez F2009 | -0.20 | 0.47 | 2.03 | 0.08 | 0.10 | 1.00 |
| G-138 | Mutus*2//TAM200/Turaco | -0.38 | 0.54 | 3.08 | 0.13 | -0.08 | 1.54 |
| G-139 | Mutus*2//Haril #1 | -0.13 | 0.19 | 1.06 | 0.13 | -0.08 | 1.54 |
| G-140 | Mutus*2/Haril #1 | -0.18 | 0.15 | 1.30 | 0.11 | -0.04 | 1.22 |
| G-141 | Quaiu/Becard//Becard | -0.45 | 0.14 | 3.10 | 0.12 | -0.07 | 1.63 |
| G-142 | Check Lehmi | 0.47 | -0.21 | 3.21 | 0.25 | -0.14 | 2.95 |
| **For Powdery mildew** | | | | | | | |
|  | Malan-16-17- (E1) | -0.47 | -0.69 |  |  |  |  |
|  | Kukumseri-16- (E2) | -0.31 | 0.19 |  |  |  |  |
|  | Palampur-16-17- (E3) | -0.22 | 0.64 |  |  |  |  |
|  | Palampur-17-18- (E4) | 1.00 | -0.13 |  |  |  |  |
| **For Yellow rust** | | | | | | | |
|  | Kukumseri 2016 (En1) |  |  |  | -0.70 | 0.58 |  |
|  | Keylong 2017 (En2) |  |  |  | 1.00 | 0.18 |  |
|  | Malan-16-17 (En3) |  |  |  | -0.30 | -0.76 |  |
